# RRNPP-type quorum-sensing systems regulate solvent formation, sporulation and cell motility in *Clostridium saccharoperbutylacetonicum*

**DOI:** 10.1186/s13068-020-01723-x

**Published:** 2020-05-08

**Authors:** Jun Feng, Wenming Zong, Pixiang Wang, Zhong-Tian Zhang, Yanyan Gu, Mark Dougherty, Ilya Borovok, Yi Wang

**Affiliations:** 1 Department of Biosystems Engineering, Auburn University, 350 Mell Street, Auburn, AL 36849 USA; 2 School of Engineering, Anhui Agricultural University, Hefei, 230036 China; 3 School of Molecular Cell Biology and Biotechnology, The George S. Wise Faculty of Life Sciences, Tel Aviv University, Ramat Aviv, 6997801 Tel Aviv, Israel; 4 Center for Bioenergy and Bioproducts, Auburn University, Auburn, AL 36849 USA

**Keywords:** *Clostridium saccharoperbutylacetonicum*, Butanol, Acetone–butanol–ethanol (ABE), RRNPP-type quorum-sensing systems, Signaling peptide precursor, Cell motility, Sporulation, CRISPR–Cas9

## Abstract

**Background:**

*Clostridium saccharoperbutylacetonicum* N1-4 (HMT) is a strictly anaerobic, spore-forming Gram-positive bacterium capable of hyper-butanol production through the well-known acetone–butanol–ethanol fermentation process. Recently, five putative RRNPP-type QSSs (here designated as QSS1 to QSS5) were predicted in this bacterial strain, each of which comprises a putative RRNPP-type regulator (QssR1 to QssR5) and a cognate signaling peptide precursor (QssP1 to QssP5). In addition, both proteins are encoded by the same operon. The functions of these multiple RRNPP-type QSSs are unknown.

**Results:**

To elucidate the function of multiple RRNPP-type QSSs as related to cell metabolism and solvent production in N1-4 (HMT), we constructed *qssR*-deficient mutants ΔR1, ΔR2, ΔR3 and ΔR5 through gene deletion using CRISPR–Cas9 and N1-4-dcas9-R4 (with the QssR4 expression suppressed using CRISPR–dCas9). We also constructed complementation strains by overexpressing the corresponding regulator gene. Based on systematic characterization, results indicate that QSS1, QSS2, QSS3, and QSS5 positively regulate the *sol* operon expression and thus solvent production, but they likely negatively regulate cell motility. Consequently, QSS4 might not directly regulate solvent production, but positively affect cell migration. In addition, QSS3 and QSS5 appear to positively regulate sporulation efficiency.

**Conclusions:**

Our study provides the first insights into the roles of multiple RRNPP-type QSSs of *C. saccharoperbutylacetonicum* for the regulation of solvent production, cell motility, and sporulation. Results of this study expand our knowledge of how multiple paralogous QSSs are involved in the regulation of essential bacterial metabolism pathways.

## Introduction

Quorum-sensing allows bacterial cells to regulate gene expression in response to the variation in cell-population density. Bacterial cells produce extracellular chemical signals (small molecules or peptides), which could accumulate in a local environment to critical levels and thereby regulate expression of specific pathways in response to population density [1–3]. Both Gram-positive and Gram-negative bacteria have been reported to use quorum sensing for communication to regulate various physiological activities. However, the mechanisms for quorum sensing in Gram-positive and Gram-negative bacteria are usually different. In general, Gram-negative bacteria use acylated homoserine lactones as autoinducers for quorum sensing, while Gram-positive bacteria use processed oligo-peptides to communicate with each other [4, 5]. It is essential to elucidate the effects of quorum-sensing systems (QSSs) in the bacterial host in order to understand the social biology of the bacteria, treat the relevant infectious diseases, and enhance the production of desirable metabolites.

Acetone–butanol–ethanol (ABE) fermentation with solventogenic clostridia has been a well-known industrial process since the early twentieth century. The ABE fermentation has two phases, acidogenesis and solventogenesis [6, 7]. During the acidogenesis phase, fatty acids including acetic acid and butyric acid are synthesized to maximize ATP generation to support active cell growth. With continuous accumulation of fatty acids, extracellular acids will diffuse back into cells, which may inhibit cell metabolism and cause acid crash. To avoid such an outcome, the cell will switch metabolism from acidogenesis to solventogenesis during which acids are re-assimilated and solvents (ABE) are produced [7, 8]. However, the mechanism to shift metabolism from acidogenesis to solventogenesis is not well understood. Nevertheless, tremendous efforts have been invested by researchers to improve solvent production in various solventogenic clostridial strains through metabolic engineering [9–11]. However, limited success has been achieved so far, largely due to our poor understanding of the regulation of cell metabolism, especially as it relates to solvent production.

To date, there are only a small number of reports concerning QSSs in solventogenic clostridial species [7, 12, 13]. There are two main types of QSSs in solventogenic clostridia: the *agr*-like [12, 14] and the RRNPP-type [13]. The *agr* (accessory gene regulator) system was first discovered in *Staphylococcus* [15] and is controlled by an auto-inducing peptide (AIP). AIP is synthesized and secreted during cell growth and activates specific gene expression when accumulated to certain levels. The core mechanism for the production and sensing of AIP is accomplished by genes organized in the *agrBCDA* operon. When extracellular AIP accumulates to certain concentrations, it will be sensed by the histidine kinase AgrC, resulting in the phosphorylation of the response regulator AgrA. Subsequently, the phosphorylated AgrA will regulate the expression of target genes or pathways [16, 17]. The RRNPP (formerly known as RNPP) QSSs were named after the well-studied peptide-sensing regulatory proteins: **R**ap, **R**gg, **N**prR, **P**lcR, and **P**rgX) [5, 18, 19]. Phylogenetic analysis suggested that all regulatory proteins were derived from a common ancestor and in fact form a single family with conserved features [20]. RRNPP family members are characterized by the presence of tetratricopeptide repeats (TPRs) that are responsible for promoting protein–protein (more specifically, protein–peptide) interactions [20, 21]. RRNPP-type QSSs comprised quorum-sensing regulators and cognate signaling peptides. These two above components can directly interact with each other and regulate relevant cell metabolism. The signaling peptide, which is derived from the C-terminus of the signaling peptide precursor through proteolysis during secretion, is imported into the cell again by the oligopeptide permease (Opp) transport system. The mature signaling peptide can interact with the regulator protein, thereby activating or inhibiting relevant cell metabolism.

Both the *agr*- and RRNPP-type QSSs have been investigated in *C. acetobutylicum*, the model microorganism for ABE fermentation. In 2012, Steiner and co-workers observed that the *arg* QSS participated in the regulation of sporulation and granulose formation in *C. acetobutylicum* [12]. Recently, the same group studied multiple RRNPP-type QSSs in *C. acetobutylicum* using ClosTron for inactivation of genes encoding these systems [13]. Their results suggested that seven of the eight RRNPP-type QSSs (QssA–H) affected solvent formation, and it was inferred that QssB was involved in the regulation of sporulation and early solventogenesis. Interestingly, both types of QSSs (*agr* and RRNPP) in various solventogenic clostridial strains are encoded by multiple genomic loci, leaving many questions regarding their cooperation and co-regulation.

*Clostridium saccharoperbutylacetonicum* N1-4 (HMT) is well-known as a hyper-butanol producer [22]. In 2007, Kosaka and co-workers speculated that a QSS might be participating in regulation of the solvent metabolism of N1-4 (HMT) [7]. Recently, Kotte and co-workers predicted multiple QSSs in *C. saccharoperbutylacetonicum* while primarily investigating RRNPP-type QSSs in *C. acetobutylicum* [13]. The elucidation of QSSs function in N1-4 (HMT) is highly desirable for understanding the mechanism of solventogenesis and further improving host solvent production through metabolic engineering. Due to the lack of highly efficient genetic engineering tools, it was not possible to conduct an in-depth QSSs investigation nor the relevant strain regulatory mechanisms. Recently, our group has developed highly efficient genome editing tools for the N1-4 (HMT) strain based on the CRISPR–Cas9 system [23]. With these versatile genetic tools, the RRNPP-type QSSs in N1-4 (HMT) were systematically investigated through precise and clean gene deletion. Our study revealed for the first time that multiple RRNPP-type QSSs of *C. saccharoperbutylacetonicum* play important roles in solvent production, cell motility, and sporulation. Moreover, the results of this study expand our knowledge about the function of bacterial QSSs related to essential pathways of cellular metabolism.

## Results and discussion

### Construction of mutant strains

Recently, multiple RRNPP-type QSSs in *C. acetobutylicum* ATCC 824 have been characterized by Kotte and co-workers [13]. The function of these QS systems was investigated through the insertional inactivation of the corresponding regulator gene using ClosTron technology [13]. The eight RRNPP-type QSSs (QssA to QssH) have been reported to play important roles in the life cycle of *C. acetobutylicum* ATCC 824. Interestingly, no such similar QSSs have been identified in another prominent ABE-producing strain, *C. beijerinckii* NCIMB 8052; while in *C. saccharoperbutylacetonicum* N1-4 (HMT), five putative RRNPP-type QSSs have been predicted [13]. Thus, in this study we aimed to elucidate the function of these QSSs in *C. saccharoperbutylacetonicum* as related to its hyper-butanol production phenotype.

**Fig. 1:**
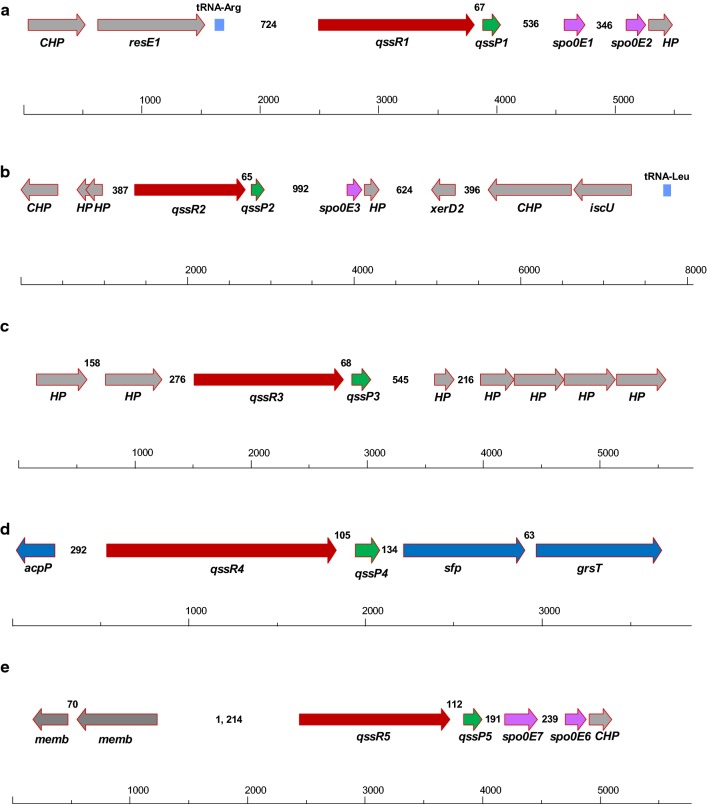
Schematic representation of the five genomic regions encoding putative RRNPP-type quorum-sensing systems in *Clostridium saccharoperbutylacetonicum* N1-4 (HMT). Genomic regions were designated as QSS1 (**a**), QSS2 (**b**), QSS3 (**c**), QSS4 (**d**) and QSS5 (**e**), respectively. Gene names are the following: *HP*, hypothetical protein; *CHP*, conserved hypothetical protein; *memb*, membrane-associated protein; *spo0E*, Spo0E-like sporulation regulatory protein; *qssR1*-*5*, putative RRNPP-type regulator (QssR1 to QssR5); *qssP1*-*5*; cognate signaling peptide precursor (QssP1 to QssP5); *resE1*, sensor histidine kinase ResE; *xerD2*, site-specific recombinase XerD; *iscU*, NifU-like protein involved in Fe-S cluster formation; *acpP*, acyl carrier protein; *sfp*, phosphopantetheine-protein transferase; *grsT*, oleoyl-(acyl-carrier-protein) hydrolase. The number indicates the nucleotide base pairs between two genes. The arrows in red represent the genes that have been deleted in this study

Figure 1 shows the arrangement of all five predicted RRNPP-type QSSs within the N1-4 (HMT) genome, designated as QSS1, QSS2, QSS3, QSS4, and QSS5, respectively. As in the case of *C. acetobutylicum*, each RRNPP-type QS system in N1-4 (HMT) comprises two predicted functional protein units: an RRNPP regulator (QssR1, QssR2, QssR3, QssR4, or QssR5) and a putative cognate signaling peptide precursor (QssP1, QssP2, QssP3, QssP4, or QssP5).

To elucidate the function of RRNPP-type QS systems in N1-4 (HMT), their regulator genes were deleted using the CRISPR–Cas9 system recently developed in our lab [23]. Four of the five *qssR* genes (*qssR1*, *qssR2*, *qssR3*, and *qssR5*) were successfully deleted (Additional file 1: Figure S2), and the resulting mutants were designated as ΔR1, ΔR2, ΔR3 and ΔR5, respectively. Interestingly, we failed to delete *qssR4* (Cspa_c29260) despite multiple attempts using different gRNAs and homology arms of various lengths (data not shown). In order to study the function of QssR4, a CRISPR–dCas9 vector was constructed and employed to inhibit the expression of *qssR4*. The resulting strain was named N1-4-dcas9-R4 [24]. There are two genes immediately downstream of the *qssR4*-*qssP4* locus, the proximal one is noted as phosphopantetheine-protein transferase (Cspa_c29240), while the distant one encodes a putative oleoyl-(acyl-carrier-protein) hydrolase (Cspa_c29230). The homologues of these enzymes are reported to participate in fatty acid biosynthesis [25, 26]. Therefore, we hypothesize it is likely that QSS4 plays an essential role in regulation of the fatty acid biosynthesis pathway and/or other crucial metabolic pathways in *C. saccharoperbutylacetonicum.* It is for this reason that *qssR4* cannot be deleted.

### Complementation of the *qssR* gene function

To better understand the regulatory role of RRNPP-type QSSs, we overexpressed the individual *qssR* gene on a plasmid in the corresponding *qssR*-deleted mutant thus fulfilling the complementation of a gene deletion. The *qssR* gene expression was driven by the *cat1* gene promoter from *C. tyrobutyricum* ATCC 25755 [27] and carried on the plasmid pMTL82151. The recombinant plasmid construct was transformed into the corresponding *qssR*-gene-deleted mutant, thus generating a set of four strains (ΔR1-R1, ΔR2-R2, ΔR3-R3, and ΔR5-R5). Moreover, the pMTL-*qssR4* plasmid (*qssR4* under control of the *cat1* promoter) was transformed into the wild-type (WT) N1-4 (HMT) strain to investigate the possible effect of *qssR4* overexpression as a function of WT phenotype.

The expression of *qssR* genes in various recombinant strains compared to WT phenotypes was investigated in three different media, P2, TGY and PG (Additional file 1: Figures S3–S5). Overall, results indicated that the medium had little impact on gene expression trend (that is, the relative expression (higher or lower) in the recombinant strain compared to that in WT), but had a significant impact on absolute expression levels of the particular *qssR* gene. Briefly, the expression levels of *qssR1*, *qssR2*, *qssR4* and *qssR5* in strains ΔR1-R1, ΔR2-R2, N1-4-R4 and ΔR5-R5, respectively, were all higher than those in the WT strain. For instance, expression of *qssR1* in ΔR1-R1 was increased by 287- to 560-fold over WT depending on different media (Additional file 1: Figures S3–S5). Interestingly, the increase in expression level of *qssR2* in ΔR2-R2 (compared to that in WT) varied remarkably in different media, by 49-fold in P2 medium (Additional file 1: Figure S3), 775-fold in PG medium (Additional file 1: Figure S5), and 25,260-fold in TGY medium (Additional file 1: Figure S4). While for *qssR4* and *qssR5*, the increase in expression levels (in strains N1-4-dcas9-R4/pMTL-*qssR4* and ΔR5-R5, respectively) was only several fold (less than ninefold) in all the media (Additional file 1: Figures S3–S5). On the other hand, the expression level of *qssR3* was lower in ΔR3-R3 than in the WT (from 5.4 to 46% less). Possibly, the native promoter of *qssR3* is much stronger than the *cat1* promoter that was used for the complementation expression. Results also indicated that the CRISPR–dCas9 system functioned well with the N1-4-dcas9-R4 strain. The *qssR4* expression was inhibited 89.8% in P2 medium, 54.3% in TGY medium and 86.4% in PG medium, respectively, as compared to that in WT (Additional file 1: Figures S3–S5).

### RRNPP-type QSSs regulate solvent formation

**Fig. 2:**
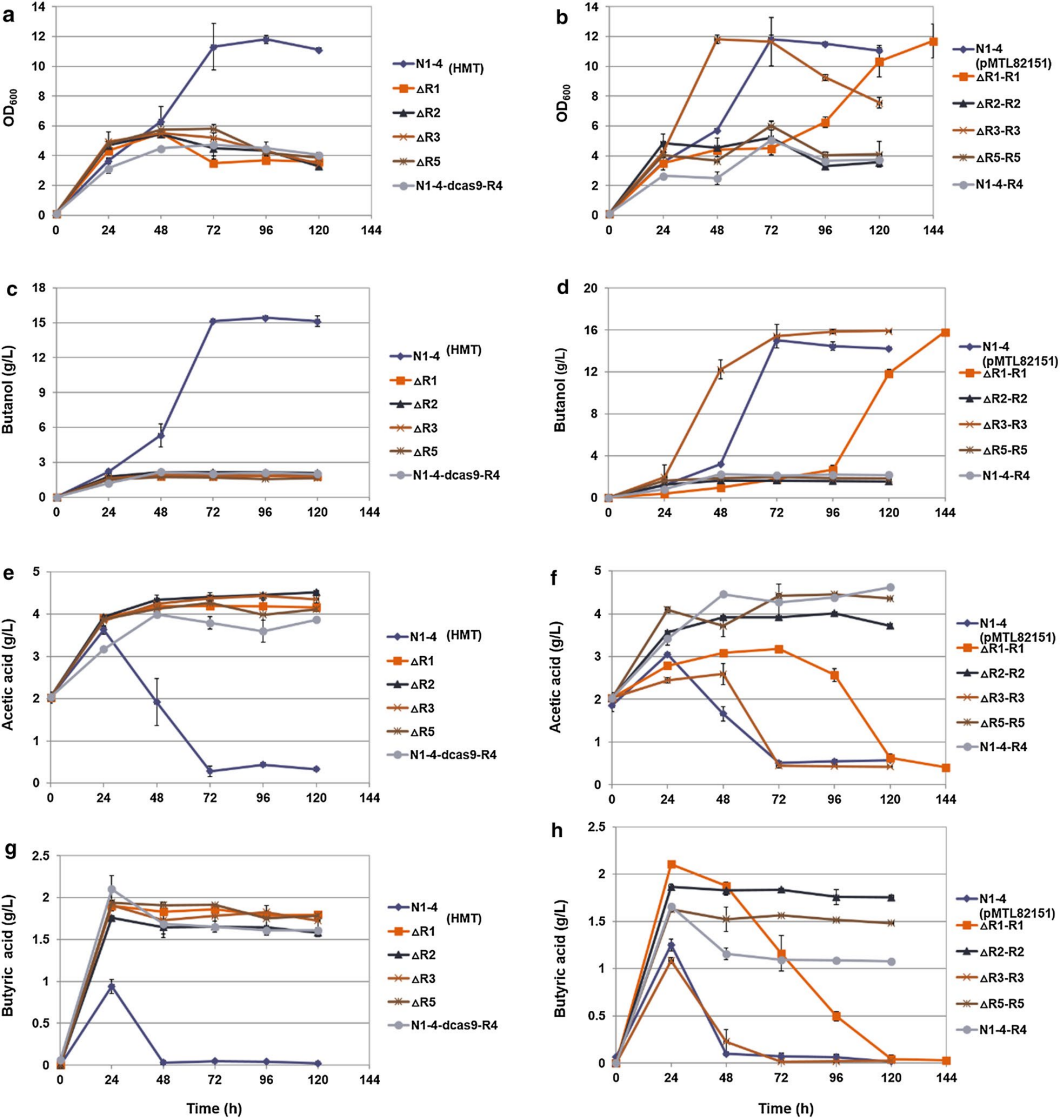
Fermentation results in small-scale serum bottles with *Clostridium saccharoperbutylacetonicum* N1-4 (HMT) and various mutant strains. Left panel (**a**, **c**, **e** and **g**): results for ∆R1, ∆R2, ∆R3, ∆R5, N1-4-dcas9-R4 as compared to the WT N1-4 (HMT). Right panel (**b**, **d**, **f** and **h**): results for ∆R1-R1, ∆R2-R2, ∆R3-R3, ∆R5-R5, N1-4-R4 as compared to N1-4 (pMTL82151). **a**, **b** Cell growth profiles; **c**, **d** butanol; **e**, **f** acetic acid; **g**, **h** butyric acid. The reported value is mean ± SD

In order to investigate the effect of RRNPP-type QSSs on solvent production in *C. saccharoperbutylacetonicum*, fermentations were first carried out in small-scale serum bottles (Fig. 2). Interestingly, all QssR-deficient mutant strains, ΔR1, ΔR2, ΔR3, and ΔR5, as well as N1-4-dcas9-R4 (with a strong repression in *qssR4* expression), exhibited so-called “acid crash” with negligible solvent production (Fig. 2a, c, e, f) [28]. The mutant strains grew well at the beginning of the fermentation and exhibited similar growth and acidogenesis profiles compared to the WT strain. However, the mutant strains failed to switch to solventogenesis. Consequently, acids were not efficiently re-assimilated and accumulated to high levels in the medium, leading to severe inhibition on cell growth and unsuccessful solvent production.

For the complementation strain, ΔR3-R3 exhibited similar profiles as the control for acid production, reassimilation, and solvent production. The N1-4(pMTL82151) strain served as the control for the fermentation. Cell growth in ΔR3-R3 was even slightly faster than the N1-4(pMTL82151) control; and final butanol production was slightly higher than the control, as well (Fig. 2b, d, f, h). In addition, ΔR1-R1 exhibited delayed profiles compared to the control for cell growth, acid reassimilation, and butanol production. However, by the end of the fermentation it reached similar levels as the control for maximum cell optical density and butanol production, although this took a much longer time. However, other complementation mutants, including ΔR2-R2 and ΔR5-R5, as well as N1-4-R4 (with the overexpression of *qssR4* in WT), showed acid crash phenomenon similar to QssR-deficient mutants (Fig. 2b, d, f, h).

**Fig. 3:**
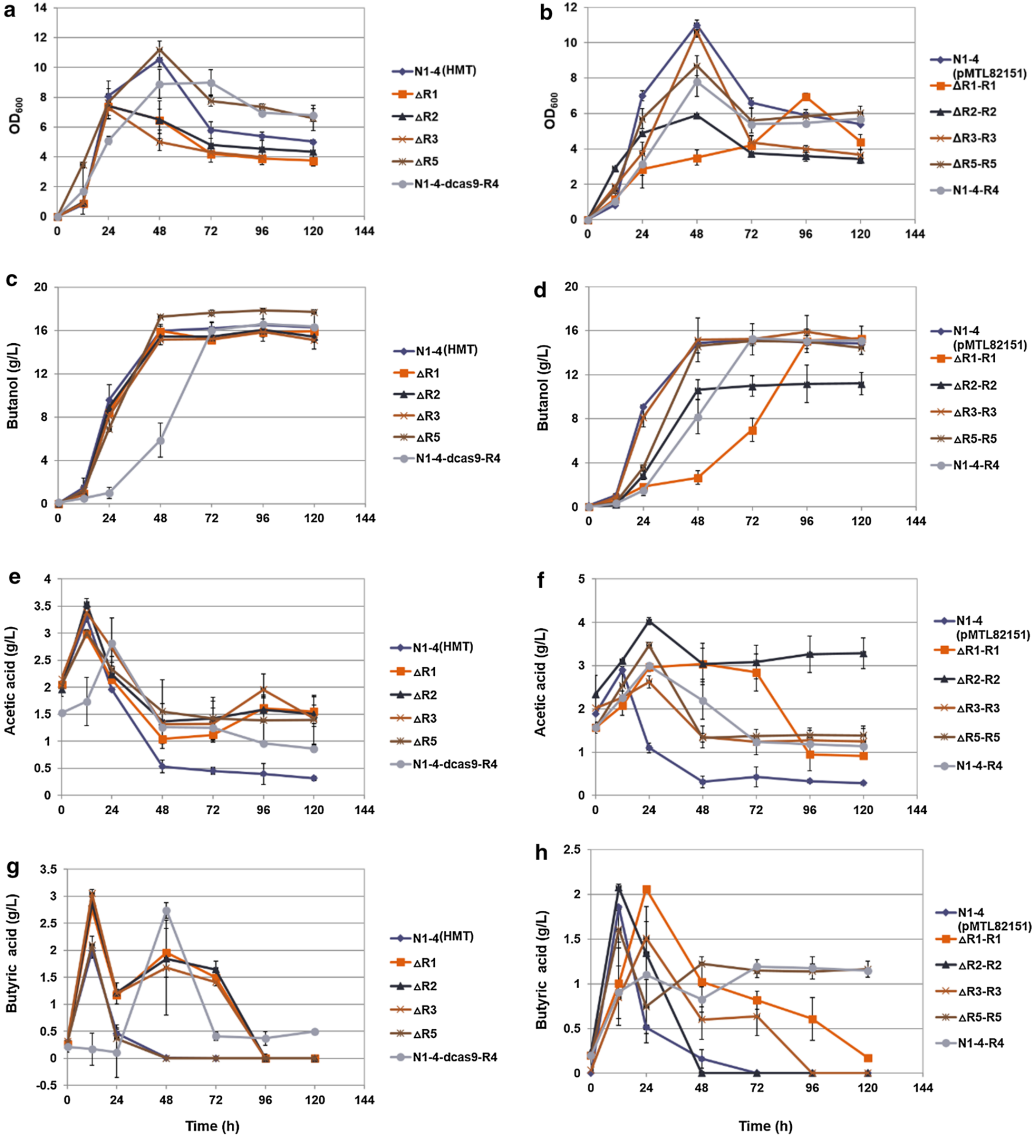
Fermentation results in 500-mL bioreactors with pH control using *Clostridium saccharoperbutylacetonicum* N1-4 (HMT) and various mutant strains. Left panels (**a**, **c**, **e** and **g**): results for ∆R1, ∆R2, ∆R3, ∆R5, N1-4-dcas9-R4 as compared to the WT N1-4 (HMT). Right panels (**b**, **d**, **f** and **h**): results for ∆R1-R1, ∆R2-R2, ∆R3-R3, ∆R5-R5, N1-4-R4 as compared to N1-4 (pMTL82151). **a**, **b** Cell growth profiles; **c**, **d** butanol; **e**, **f** acetic acid; **g**, **h** butyric acid. The reported value is mean ± SD

To prevent acid crash caused by acid accumulation in the medium, we subsequently performed the fermentation in 500-mL, pH-controlled bioreactors (pH ≥ 5.0). Initially, we started the fermentation with 5% (v/v) inoculation using the preculture of OD_600_ = ~ 0.8. However, the mutant strains did not grow well. Consequently, OD_600_ of the inoculum preculture was raised to ~ 1.2 before fermentation. As shown in Fig. 3(left panel), compared to fermentation in the serum bottle, fermentation in the bioreactor improved significantly. Although most of the QssR-deficient mutants demonstrated slightly inferior capability for acid reassimilation than the control, each re-assimilated acids adequately and produced similar levels of butanol. In fact, the ΔR5 strain produced slightly higher levels of butanol than the control. The N1-4-dcas9-R4 strain demonstrated a delayed metabolism compared to the control, with cell growth, acid production and reassimilation, and butanol production all slower. However, by the end of the fermentation, the N1-4-dcas9-R4 strain produced approximately the same amount of butanol as the control.

On the other hand, when fermentation with the complementation strain is compared to the control N1-4(pMTL8215), ΔR1-R1 had delayed and inhibited cell growth, as well as lesser capability for acid reassimilation and much delayed butanol production (right panel in Fig. 3). However, by the end of the fermentation (> 96 h), the strain produced a level of butanol similar to the control. The ΔR2-R2 stain had 46% less cell growth than the control, diminished acetate reassimilation (interestingly, the generated butyrate was efficiently re-assimilated), and decreased butanol production (25% less than the control). ΔR3-R3 and ΔR5-R5 exhibited similar cell growth and butanol production (both production rate and level) as the control. The N1-4-R4 strain (with the overexpression of R4) exhibited decreased cell growth and lesser capability for acid reassimilation; and its butanol production was also delayed. Nevertheless, by the end of fermentation, the N1-4-R4 strain achieved a maximum level similar to the control (although it took about 24 h longer). Further, we observed that it was difficult to cultivate the N1-4-dcas9-R4 strain with an inoculation ratio of 5% in TGY medium, so we had to increase the inoculation ratio for the cultivation to 10%. Above these observations suggest that *qssR4* is essential for *C. saccharoperbutylacetonicum*, and that a strict control of *qssR4* expression at the appropriate level is crucial for normal cell metabolism.

**Fig. 4:**
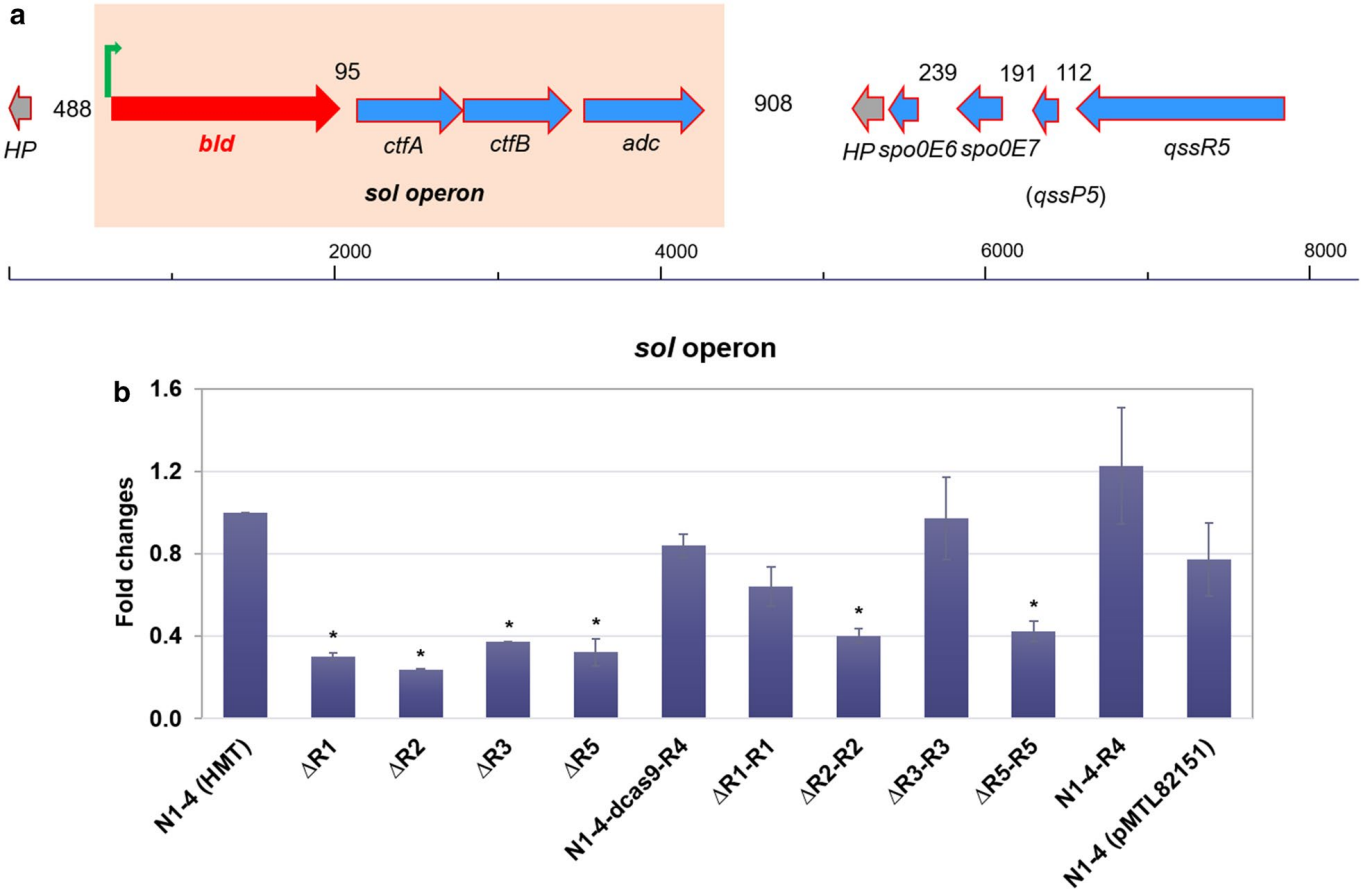
Schematic representation of the *C. saccharoperbutylacetonicum* N1-4 (HMT) genomic region comprising both the *sol* operon (shown within a rectangle) and QSS5 genes (**a**) and transcriptional analyses of the *sol* operon (*bld* was used for the qRT-PCR test as a representative) in N1-4 (HMT) and various mutants (**b**). Gene names are the following: *bld*, butyraldehyde dehydrogenase (Cspa_c56880); *ctfA*, butyrate-acetoacetate CoA-transferase subunit A (Cspa_c56890); *ctfB*, butyrate-acetoacetate CoA-transferase subunit B (Cspa_c56900); *adc*, acetoacetate decarboxylase (Cspa_c56910); other gene names as shown in Fig. 1. Bacterial RNAs were extracted from the cell culture after 24 h cultivation in P2 medium. The reported value is mean ± SD. The asterisk indicates that the corresponding gene expression level in that particular strain was significantly different from the WT strain (*P* < 0.05)

The *sol* operon plays essential roles in the clostridial solventogenesis [29, 30]. This operon should be appropriately induced and expressed when cell metabolism shifts from acidogenesis to solventogenesis [31, 32]. The *sol* operon in N1-4 (HMT) consists of four genes in the order of *bld*, *ctfA*, *ctfB* and *adc* (Fig. 4). Kosaka and coworkers [5] reported that the *sol* operon in *C. saccharoperbutylacetonicum* was transcribed in a polycistronic manner and controlled by two promoters; and that the *sol* operon is highly expressed during solventogenesis. They also showed that the transcriptional repression of *sol* operon impaired solvent production in a degenerated strain DGN3-4 derived from N1-4; and the addition of substance extracted from the WT N1-4 culture supernatant could induce both *sol* operon expression and solvent production. Thus, they inferred that the transcription of the *sol* operon might be controlled by a QSS [5].

In this study, we examined the transcription of the *sol* operon in mutant strains compared to that in the WT. Cell culture was harvested from the fermentation in the serum bottle, and transcription levels were measured using qRT-PCR. Results indicate that the expression of the *sol* operon in ΔR1, ΔR2, ΔR3, ΔR5, ΔR2-R2, and ΔR5-R5 were all repressed by > 50% compared to N1-4 (HMT) (Fig. 4b), and all these strains exhibited acid crash in the serum-bottle fermentation (Fig. 2). The expression of *sol* operon in ΔR3-R3 was comparable with that in the WT. Correspondingly, solvent production level and kinetics in ΔR3-R3 were similar to the WT (Fig. 2d). The ΔR1-R1 strain exhibited a 36% decrease in its *sol* operon transcription (Fig. 4), which is consistent with fermentation results indicating that acid reassimilation and solvent production were delayed in ΔR1-R1 compared to the control (Fig. 2d, f, h). Above results suggest that expression of the *sol* operon is essential for acid reassimilation and solvent production in *C. saccharoperbutylacetonicum*. The RRNPP-type QSS regulators, including QssR1, QssR2, QssR3, and QssR5, correlate and regulate the appropriate expression of the *sol* operon. Interestingly, as demonstrated above, using seed culture of higher cell density or controlling pH to prevent acid crash, we can improve cell growth and restore acid reassimilation and solventogenesis in the mutants (Fig. 3). This indicates that the deletion of only one of the RRNPP-type regulators (either QssR1, QssR2, QssR3, or QssR5) would impair, but not completely eliminate, the cell capability for acid reassimilation and solvent production. Thus, we infer that the four regulators mentioned above might have a synergistic effect on induction of the *sol* operon expression for acid reassimilation and solvent production.

On the other hand, expression of the *sol* operon in N1-4-dcas9-R4 and N1-4-R4 is at a level similar and slightly higher (by 23%), respectively, than in WT. However, these two strains still exhibited ‘acid crash’ in the fermentation. Therefore, we conclude that the Qss4 system might not directly regulate the expression of the *sol* operon, rather, it might be essential for other key cell metabolism. Thus, either the repression or overexpression of the *qssR4* gene could impair cell regular metabolism for acid reassimilation and solvent production.

### RRNPP-type QSSs regulate cell motility

**Fig. 5:**
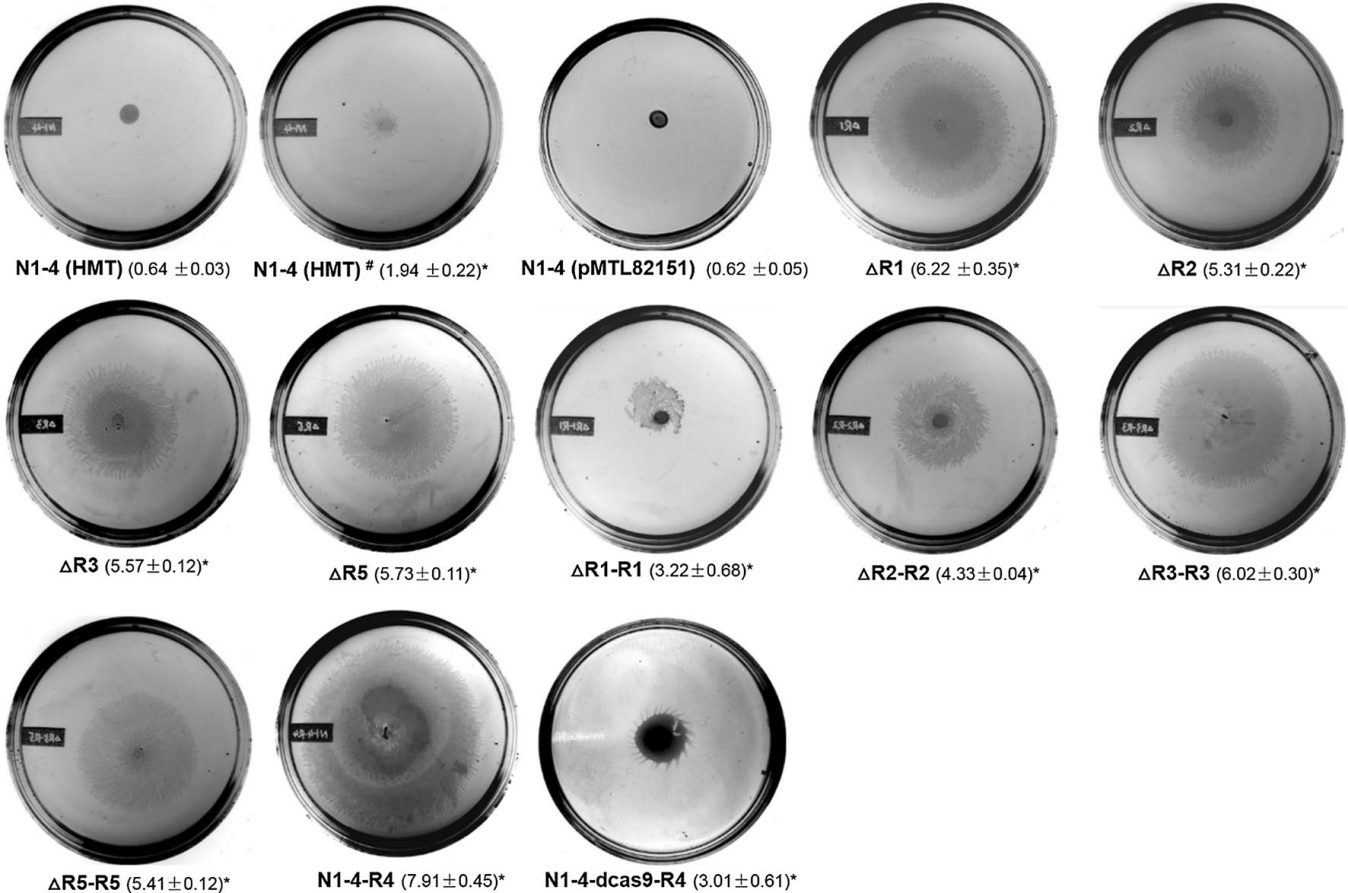
The cell motility in *Clostridium saccharoperbutylacetonicum* N1-4 (HMT) and various mutants, represented by the diameter of cell migration on soft agar plates. The WT N1-4 (HMT) strain demonstrated two different motility sizes: N1-4 (HMT) and N1-4 (HMT)^#^. The cell culture was grown in TGY medium until the OD_600_ reached ~ 0.8. Two microliters of the cell culture was spotted onto the center of the TGY plate containing 0.5% agar. The diameter of the cell migration was measured after 12 h of cultivation at 35 °C anaerobically. The reported value (the value in the parentheses underneath of each plate image, in centimeter) is mean ± SD. The asterisk indicates that the corresponding cell motility of that particular strain was significantly different from the WT strain (*P* < 0.05)

Cell motility assays were performed using soft agar plates. As shown in Fig. 5, the WT strain had two different phenotypes: one had no cell motility (N1-4 (HMT)), while the other one showed a little bit cell motility (N1-4 (HMT)^#^). Herman and co-workers observed three distinct colony morphologies from the same stock of N1-4 (HMT): type I, type R and type S. Type R can change to type I at high frequency and unpredictability, and thus authors predicted that the phenotype conversion might be caused by unclear epigenetic influences [33]. Consequently, we suggest that different cell motility phenotypes from the WT strain are related to their subtype conversions. However, the other control strain of this study, N1-4 (pMTL82151), showed no cell motility at all. All the mutant strains except for N1-4-dcas9-R4 exhibited between a 1.7-fold to 5.0-fold increase in cell motility compared to the control N1-4 (HMT) and N1-4 (HMT)^#^, respectively (expressed as the diameter of a swarm ‘circle’ of migrating cells on the plate). Although the cell migration of N1-4-dcas9-R4 was higher than the control strain N1-4 (HMT), cell motility appeared comparable to that of the N1-4 (HMT)^#^ strain with only slight cell migration observed.

**Fig. 6:**
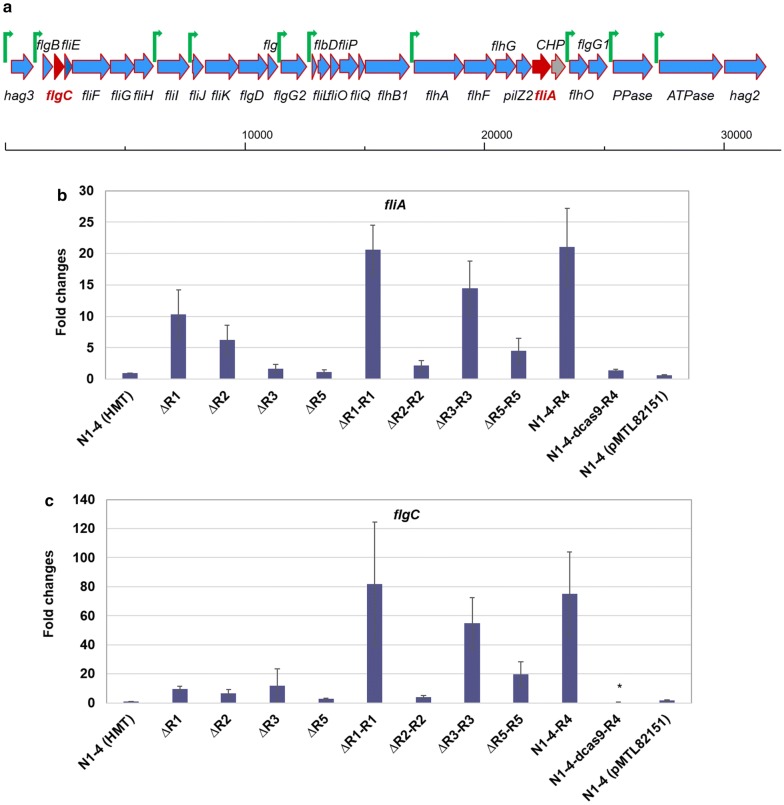
Schematic representation of the *Clostridium saccharoperbutylacetonicum* N1-4 (HMT) genomic region comprising genes (from Cspa_c45260/*hag3* to Cspa_c44960/*hag2*) of the flagellar regulon; *flgC* and *fliA* genes are shown in red (**a**) and transcriptional analyses of *fliA* (**b**) and *flgC* (**c**) using qRT-PCR in N1-4 (HMT) and various QSS mutants. RNA samples were extracted from the cell culture after 12 h cultivation in TGY medium. The reported value is mean ± SD. The asterisk indicates that the corresponding gene expression level in that particular strain was significantly different from the WT strain (*P* < 0.05). Green upwards arrows with tip rightwards (**a**) indicate promoters of the predicted operons

Bacterial flagella are motility organelles for cell locomotion [34, 35]. Nearly 50 genes control the formation, regulation and function of these flagella [36–38]. Almost half of these genes contribute to the physical structure of the flagella, whereas other genes have regulatory or auxiliary functions [36]. Figure 6a shows the genomic organization of the large gene cluster associated with flagellar synthesis in strain N1-4 (HMT), where 30 genes make up ten putative operons. To elucidate the regulation of these flagellum genes as related to RRNPP-type QSSs, two genes, *flgC* (Cspa_c45240) and *fliA* (Cspa_c45030), were selected and their expression levels quantified with qRT-PCR. It has been reported that *flgC* encodes the flagellar basal-body rod protein; while *fliA* encodes for the alternative RNA polymerase sigma factor (FliA/FlgM-family), assumed to positively regulate the flagellar synthesis [39]. Transcriptional activity of both *fliA* (Fig. 6b) and *flgC* (Fig. 6c) in all mutant strains (including both the QssR-deficient strains and their complementation strains) were > 70% higher than WT N1-4 (HMT), except for the *flgC* gene in N1-4-dcas9-R4 (with a suppressed *qss4*) which showed a nearly 60% decrease of expression when compared to the WT. Although the transcription levels of *fliA* and *flgC* are not perfectly consistent with corresponding cell motility as demonstrated in soft agar plating assays, the general trends were agreeable (Figs. 5 and 6). Overall, results indicated that the upregulation of *fliA* and *flgC* in most of the mutants would lead to augmented synthesis of flagella and thereby increased cell motility. The transcription levels of *fliA* and *flgC* in ΔR1-R1 increased by 21-fold and 82-fold above the WT, respectively. However, the cell motility of ΔR1-R1 appeared impaired and exhibited an irregular shaped cell swarm compared to ΔR1. We deduce that the overexpression of *qssR1* might have disturbed the tightly controlled flagellar synthesis, which resulted in an irregular shaped cell motility. The transcription level of *fliA* in N1-4-dcas9-R4 was 38% higher than that of the WT, while expression of *flgC* was 59% lower than the WT. Considering cell motility results with respect to the expression levels of *fliA* and *fliC* in both N1-4-dcas9-R4 and N1-4-R4 strains, we tentatively conclude that the QSS4 system might have positive effects on flagella synthesis and cell motility.

As demonstrated in ΔR1, ΔR2, ΔR3 and ΔR5, the deletion of any *qssR* gene increased cell motility (Fig. 5) as well as expression levels of both *fliA* and *flgC* (Fig. 6b, c). Exceptionally, the increase of expression of *fliA* and *flgC* in mutant ΔR5 was only 14% and 170%, respectively, which was much less remarkable than upregulation of these genes in other strains. Therefore, it appears that deleting *qssR* genes leads to upregulation of flagellar synthesis. Nevertheless, the simultaneous increase in cell motility and expression levels of both *fliA* and *flgC* in corresponding complementation strains ΔR1-R1, ΔR2-R2, ΔR3-R3 and ΔR5-R5 have also been observed. Because this result appears contradictory, we additionally quantified the expression levels of *qssR1*, *qssR2*, *qssR3* and *qssR5* (Additional file 1: Figure S4) as well as those of *qssP1*, *qssP2*, *qssP3* and *qssP5* (Additional file 1: Figure S6). These experiments were completed using TGY medium in all strains (obviously, expression of a given *qssR* gene could not be measured in its cognate QssR-deficient strain because of a complete deletion of this gene). Results indicate that expression levels of *qssR1*, *qssR2*, and *qssR5* in complementation strains ΔR1-R1, ΔR2-R2 and ΔR5-R5 were all significantly higher than the control (Additional file 1: Figure S4a, b) and RRNPP-type QSS consists of two proteins: the transcriptional regulator and its cognate signaling peptide precursor [13]. The intact function of the RRNPP-type QSS relies on the role of both parts. The change of expression levels in either could influence the regulation of the target genes. In ΔR1-R1, ΔR2-R2 and ΔR5-R5, transcription levels of all corresponding *qssP1*, *qssP2* and *qssP5* decreased compared to the WT strain, while in ΔR3-R3, expression levels of *qssR3* decreased significantly more than the control (Additional file 1: Figure S3c). Such repression in *qssP1*, *qssP2,* and *qssP5,* as well as *qssR3,* within these host strains could lead to malfunction of the corresponding RRNPP-type QS system. Thus, complementation strains ΔR1-R1, ΔR2-R2, ΔR3-R3 and ΔR5-R5 all exhibited similar increase in cell motility (Fig. 5). In conclusion, the QSS1, QSS2, QSS3 and QSS5 likely negatively regulate cell motility, while it appears that QSS4 positively regulates cell motility in *C. saccharoperbutylacetonicum*.

### RRNPP-type QSSs regulate the cell sporulation

RRNPP-type QSSs are reported to regulate the sporulation in *Bacillus* species and *C. difficile* [40–42]. The Rap and NprR proteins in *Bacillus* species can directly bind and dephosphorylate Spo0F, which is an intermediate phosphotransfer protein in the sporulation phosphorelay [42]. The sporulation phosphorelay thereafter modulates the phosphorylation state of Spo0A (the master regulator for sporulation), repressing cell sporulation [40]. However, contrary results have been observed in *C. difficile* in that the RstA (Rap-like protein) could actually enhance cell sporulation [40].

**Fig. 7:**
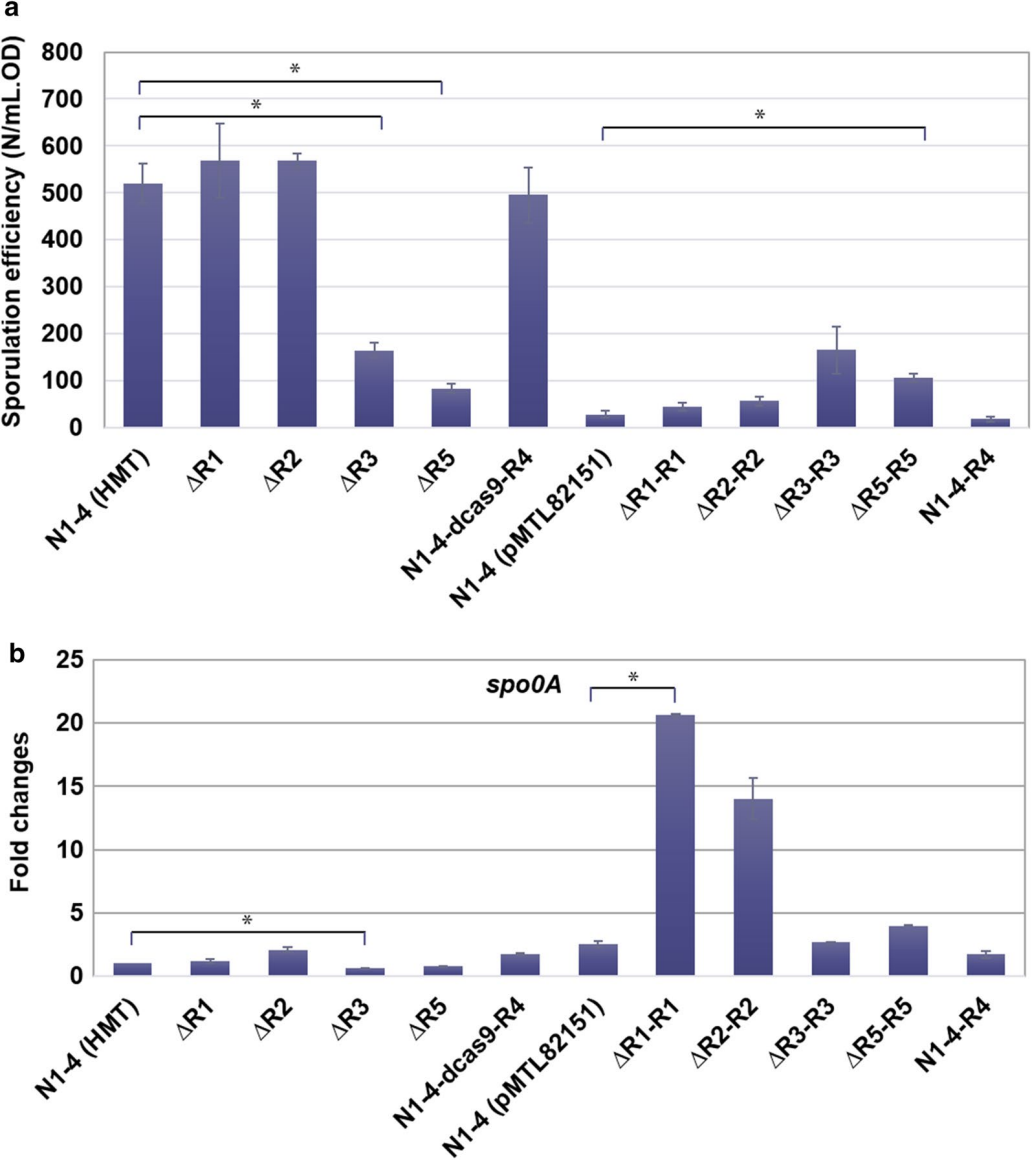
Cell sporulation efficiencies (**a**) and transcriptional analyses of *spo0A* (*Cspa_c27540*) using qRT-PCR in N1-4 (HMT) and various mutants (**b**). Bacterial RNAs were extracted from the cell culture after 24 h cultivation in PG medium. The reported value is mean ± SD. The asterisk indicates that the corresponding gene expression level in that particular strain was significantly different from the control strain N1-4 (HMT) (*P* < 0.05)

Five of the seven *spo0E*-like genes in *C. saccharoperbutylacetonicum* N1-4 (HMT) are located immediately downstream of the genes encoding QSS1, QSS2, and QSS5. Therefore, we hypothesize that the above QSSs might be involved in the regulation of cell sporulation. It is known that Spo0E is an aspartyl-phosphate phosphatase which specifically dephosphorylates the sporulation transcription factor Spo0A–P and negatively regulates the sporulation initiation pathway in order to control sporulation timing [43]. To test this hypothesis, sporulation efficiency and transcription level of *spo0A* in all strains (Cspa_c27540) was quantified (Fig. 7). We observed that the ‘empty’ pMTL82151 plasmid significantly repressed sporulation. Thus for sporulation in ΔR1, ΔR2, ΔR3, ΔR5 and N1-4-dcas9-R4, we used N1-4 (HMT) as the control, while for sporulation in ΔR1-R1, ΔR2-R2, ΔR3-R3, ΔR5-R5 and N1-4-R4, we used N1-4 (pMTL82151) as the control. Results indicate that ΔR3 and ΔR5 had significantly decreased sporulation efficiency compared to the N1-4 (HMT) strain. Corresponding complementation strains ΔR3-R3 and ΔR5-R5 had increased sporulation efficiencies compared to the N1-4 (pMTL82151) strain (Fig. 7a). Expression levels of *spo0A* in ΔR3 and ΔR5 were lower (by 37% and 22%, respectively) than that of the N1-4 (HMT) strain (Fig. 7b), which was consistent with sporulation efficiency results in these strains (Fig. 7b). The expression level of *spo0A* in ΔR5-R5 was 1.65-fold higher than that of the N1-4 (pMTL82151) strain, while the expression level of *spo0A* in ΔR3-R3 was comparable with N1-4 (pMTL82151) strain (2.67 vs 2.54). Overall, we concluded that QSS3 and QSS5 systems positively regulate sporulation; hence, the deletion of *qssR3* or *qssR5* lowered sporulation efficiency while the overexpression of them resulted in increased sporulation efficiency (although the expression level of *spo0A* in ΔR3-R3 did not demonstrate a corresponding significant increase). These results are similar to the case of *C. difficile* in which RstA protein positively regulates sporulation [40].

ΔR1 and ΔR2 strains were observed to have a slight increase in their sporulation efficiency and *spo0A* expression. Their complementation mutants ΔR1-R1 and ΔR2-R2 demonstrated minor increase in sporulation efficiency and significantly higher *spo0A* expression levels compared to the control (Fig. 7). Unlike gene expression changes in TGY and P2 mediums, the gene expressions of *qssP1* and *qssP2* from ΔR1-R1 and ΔR2-R2 in the PG medium were all significantly improved (Additional file 1: Figures S6–S8). Combined with the enhancement of *qssR1* and *qssR2* (Additional file 1: Figure S5), we conclude that the QSS1 and QSS2 were enhanced in both the ΔR1-R1 and ΔR2-R2 strains. Both the repression (through the deletion of the regulator gene) and the enhancement (through plasmid-based complementation) of QSS1 and QSS2 demonstrated similar results in terms of the cell sporulation efficiency. Thus, we were not able to draw a definitive conclusion concerning the regulation effect of QSS1 and QSS2 on cell sporulation. The expression level of *spo0A* significantly increased in both ΔR1-R1 and ΔR2-R2 while their sporulation efficiency showed only marginal improvement compared with the control N1-4 (pMTL82151). This result might be related to the significant upregulation of *spo0E*-like genes located downstream of the QSS1, QSS2 and QSS5 operons (Additional file 1: Figure S9). Spo0E has been reported to be involved in the dephosphorylation of Spo0A and is thus a negative regulator of cell sporulation [43]. The elevated expression of *spo0E1* and *spo0E3* in ΔR1-ΔR1 and ΔR2-ΔR2 might have intensified the dephosphorylation of Spo0A ~ P and thus did not result in a significantly increased sporulation efficiency (although the expression level of *spo0A* has been significantly elevated in both strains).

There were no significant changes in *spo0A* expression or sporulation efficiency in either N1-4-dcas9-R4 or N1-4-R4 when compared to the control N1-4 (HMT) or N1-4 (pMTL82151), respectively (Fig. 7). Therefore, QSS4 likely has no significant regulatory effect on cell sporulation.

Interestingly, we observed that in N1-4 (pMTL82151) the expression level of all three *spo0E*-like genes decreased and that of *spo0A* increased (Fig. 7 and Additional file 1: Figure S9). As described above, Spo0E phosphatase could negatively regulate the activity of Spo0A, the master regulator which should positively regulate the sporulation. The sporulation efficiency in N1-4 (pMTL82151) strain should be higher than the WT N1-4 (HMT) strain, based on above data. However, sporulation efficiency in N1-4 (pMTL82151) decreased significantly compared to the N1-4 (HMT) strain (Fig. 7). In fact, sporulation efficiencies in all strains bearing pMTL82151 or its derivate plasmid decreased remarkably. We speculate that the addition of antibiotics (particularly 15 μg/mL thiamphenicol) may have resulted in this phenomenon.

## Conclusions

This is the first report concerning the RRNPP-type QSSs in *C. saccharoperbutylacetonicum* N1-4 (HMT). Results indicate that RRNPP-type QS systems play significant roles in the regulation of solvent formation, cell motility and sporulation. We observed that QSS1, QSS2, QSS3, and QSS5 positively regulate the *sol* operon expression and thus solvent production, but likely negatively regulate cell motility. QSS4 might not directly regulate solvent production, but positively affects cell migration. In addition, QSS3 and QSS5 appear to positively regulate sporulation efficiency. Our study provides original insights into the possible roles of multiple RRNPP-type QSSs of *C. saccharoperbutylacetonicum* for the regulation of cell metabolism.

## Materials and methods

### Microorganisms and cultivation conditions

All the strains and plasmids used in this study are listed and described in Table 1. *C. saccharoperbutylacetonicum* N1-4 (HMT) (DSM 14923, = ATCC 27021) was obtained from DSMZ, Germany. For routine cultivation, all *C. saccharoperbutylacetonicum* strains (both WT and mutants) were grown in an anaerobic chamber (N_2_–CO_2_–H_2_ with a volume ratio of 85:10:5) at 35 °C in tryptone–glucose–yeast extract (TGY) medium [44]. When needed, clarithromycin (Cla) and thiamphenicol (Tm) were added into the TGY medium to a final concentration of 30 µg/mL and 15 µg/mL, respectively. *Escherichia coli* DH5α were used for routine plasmid propagation and maintenance. It was grown aerobically at 37 °C in Luria–Bertani (LB) medium supplemented with 100 µg/mL of ampicillin (Amp) or 34 µg/mL chloramphenicol (Cm), as needed.

**Table 1:** Strains and plasmids used in this study

Strains and plasmids	Relevant characteristics	Source or reference
Strains		
*C. saccharoperbutylacetonicum* N1-4 (HMT)	N1-4, DSM 14923 (=ATCC 27021), WT strain	DSMZ
*C. saccharoperbutylacetonicum* ΔR1	Derived from N1-4, Δ*Cspa_c00280*	This study
*C. saccharoperbutylacetonicum* ΔR2	Derived from N1-4, Δ*Cspa_c21720*	This study
*C. saccharoperbutylacetonicum* ΔR3	Derived from N1-4, Δ*Cspa_c27220*	This study
*C. saccharoperbutylacetonicum* N1-4-dcas9-R4	N1-4 with the expression of pYW-19d-*qssR4*	This study
*C. saccharoperbutylacetonicum* ΔR5	Derived from N1-4, Δ*Cspa_c56960*	This study
*C. saccharoperbutylacetonicum* ΔR1-R1	ΔR1 strain with the expression of pMTL-*qssR1*	This study
*C. saccharoperbutylacetonicum* ΔR2-R2	ΔR2 strain with the expression of pMTL-*qssR2*	This study
*C. saccharoperbutylacetonicum* ΔR3-R3	ΔR3 strain with the expression of pMTL-*qssR3*	This study
*C. saccharoperbutylacetonicum* N1-4-R4	N1-4 strain with the expression of pMTL-*qssR4*	This study
*C. saccharoperbutylacetonicum* ΔR5-R5	ΔR5 strain with the expression of pMTL-*qssR5*	This study
*C. saccharoperbutylacetonicum* N1-4 (pMTL82151)	N1-4 strain with the expression of pMTL82151	This study
*Clostridium tyrobutyricum* ATCC 25755	ATCC 25755 (= KCTC 5387)	ATCC
*E. coli* DH5α	F^−^, φ80d*lac*ZΔM1, Δ(*lacZYA*-*argF*)U169, *deoR*, *recA*1, *endA*1, *hsdR*17(r_k_^−^, m_k_^+^), *phoA*, *supE*44, λ^−^*thi*-1, *gyrA*96, *relA*1	NEB
Plasmids		
pYW34	CAK *ori*, Amp^r^, Erm^r^, Plac::Cas9, gRNA	[24]
pMTL82151	pBP1 *ori*, *catP*, ColE1, *tra*	[47]
pYW19d-*BseR*I	CAK *ori*, Amp^r^, Erm^r^, Pthl::dCas9, gRNA	[48]
pYW34-∆*qssR1*	Derived from pYW34, J23119::20-nt gRNA targeting *Cspa_c00280*, homology arms	This study
pYW34-∆*qssR2*	Derived from pYW34, J23119::20-nt gRNA targeting *Cspa_c21720*, homology arms	This study
pYW34-∆*qssR3*	Derived from pYW34, J23119::20-nt gRNA targeting *Cspa_c27220*, homology arms	This study
pYW34-∆*qssR5*	Derived from pYW34, J23119::20-nt gRNA targeting *Cspa_c56960*, homology arms	This study
pMTL-*qssR1*	Derived from pMTL82151, with the expression of *Cspa_c00280* under the control of *cat1* promoter from *C. tyrobutyricum* ATCC 25755	This study
pMTL-*qssR2*	Derived from pMTL82151, with the expression of *Cspa_c21720* under the control of *cat1* promoter from *C. tyrobutyricum* ATCC 25755	This study
pMTL-*qssR3*	Derived from pMTL82151, with the expression of *Cspa_c27220* under the control of *cat1* promoter from *C. tyrobutyricum* ATCC 25755	This study
pMTL-*qssR4*	Derived from pMTL82151, with the expression of *Cspa_c29260* under the control of *cat1* promoter from *C. tyrobutyricum* ATCC 25755	This study
pMTL-*qssR5*	Derived from pMTL82151, with the expression of *Cspa_c56960* under the control of *cat1* promoter from *C. tyrobutyricum* ATCC 25755	This study
pYW19d-*qssR4*	Derived from pYW19d-*BseR*I, with J23119::20-nt gRNA targeting *Cspa_c29260*	This study

### Mutant construction

In this study, the RRNPP-type regulator genes *Cspa_c00280*, *Cspa_c21720*, *Cspa_c27220*, *Cspa_c29260* and *Cspa_c56960* were named as *qssR1*, *qssR2*, *qssR3*, *qssR4* and *qssR5*, respectively (Fig. 1); their putative cognate signaling peptide genes *Cspa_c00290*, *Cspa_c21710*, *Cspa_c27230*, *Cspa_c29250* and *Cspa_c56950* were named as *qssP1*, *qssP2*, *qssP3*, *qssP4* and *qssP5*, respectively (Fig. 1). The mutant with gene deletion was screened as previously described [23]. The final plasmid-free mutant was designated as *C. saccharoperbutylacetonicum* ∆R1, ∆R2, ∆R3, and ∆R5 (the deletion of *qssR4* was unsuccessful despite numerous attempts). The mutant bearing the corresponding plasmid for complementation purpose was named as ∆R1-R1, ∆R2-R2, ∆R3-R3, or ∆R5-R5, respectively. N1-4-R4 is the recombinant strain based on WT holding the plasmid for the overexpression of *qssR4*. Similarly, N1-4-dcas9-R4 is the recombinant strain based on WT holding the pYW-19d-*qssR4* plasmid. Refer to the Additional file 1 for more details about the construction of plasmids and mutants.

### Cell motility assay

Cell culture was grown overnight in TGY medium supplemented with antibiotics when necessary. The culture was then subcultured in fresh medium until OD_600_ reached ~ 0.8. Two microliters of prepared culture was spotted in the center of TGY plates (supplementary with appropriate antibiotics) with 0.5% agar. The diameter of cell migration was measured after 12 h of cultivation. Pictures were taken with the AlphaImager^®^ HP system (Alpha Innotech, USA).

### Sporulation assay

The cell culture was grown in TGY medium until OD_600_ reached ~ 0.8. The seed culture was then inoculated into 20 mL PG medium (150 g/L mashed potato, 10 g/L glucose, 1 g/L NH_4_SO_4_ and 3 g/L CaCO_3_, pH 6.2) [45] with an inoculation ratio of 5% and subsequently cultured for 7 days in the anaerobic chamber. The PG medium was filtered by gauze before adding CaCO_3_. An appropriate amount of 1N HCl was added into the broth to remove remaining CaCO_3_ after cultivation. OD_600_ of the cell culture was determined. Then the culture was centrifuged at 4200*g* and 4 °C for 10 min. The cell pellet was washed for twice with ddH_2_O and then resuspended into 1 mL of ddH_2_O. 100 μL of the collected spores was spread onto TGY agar plates after heat treatment (80 °C for 10 min) [45]. Colonies were counted after the incubation for 2 days in the anaerobic chamber. The sporulation efficiency was calculated as *N*/mL.OD. *N* represented the number of colonies.

### Fermentation

The ABE fermentation was performed in either serum bottles or 500-mL bioreactors. For serum bottle fermentation, cell culture was cultivated in TGY medium in the anaerobic chamber until OD_600_ reached ~ 0.8. The seed culture was then inoculated into 100 mL of P2 medium (80 g/L glucose, 2 g/L yeast extract, and 6 g/L tryptone) in a 250-mL bottle with an inoculation ratio of 5%. Fermentation was performed under anaerobic conditions at 30 °C with an agitation of 150 rpm. Analytical samples were taken every 24 h. For bioreactor fermentation, the cell culture was cultivated in TGY medium in the anaerobic chamber until OD_600_ reached ~ 1.2. Thereafter, the seed culture was inoculated into 300 mL of P2 medium (80 g/L glucose, 2 g/L yeast extract, and 6 g/L tryptone) in 500-mL bioreactors (GS-MFC, Shanghai Gu Xin biological technology Co., Shanghai, China) with an inoculation ratio of 5%. Fermentation was performed at 30 °C with an agitation of 150 rpm, with pH controlled to ≥ 5.0 using 3N NaOH. Samples were withdrawn for analysis every 12 h in the first 24 h and every 24 h afterwards. Appropriate amounts of antibiotics were added into the medium when necessary.

Concentrations of butanol, acetic acid and butyric acid were measured using an Agilent 1260 Infinity HPLC system (Agilent Technologies, Santa Clara, CA) equipped with a refractive index detector (RID), and a Varian MetaCarb 87H column (Agilent Technologies, CA). The column was eluted with 5 mM H_2_SO_4_ with a flow rate of 0.6 mL/min at 25 °C [46].

## Supplementary information

**Additional file 1: Table S1.** Primers used in this study. **Table S2.** Comparative pair-alignment data of amino acid sequences of the *Clostridium saccharoperbutylacetonicum* RRNPP-type transcriptional regulators. **Table S3.** Comparative pair-alignment data of amino acid sequences of the signaling-peptide precursors of the five *Clostridium saccharoperbutylacetonicum* RRNPP-type quorum sensing systems. **Figure S1.** Schematic of two rounds of PCR to obtain the DNA fragment containing 20-nt gRNA sequence for constructing the CRISPR–Cas9 plasmid for gene deletion. **Figure S2.** Confirmation of gene deletion by colony PCR. **Figure S3.** Transcriptional analyses of *qssR1*, *qssR2*, *qssR3*, *qssR4* and *qssR5* in wild-type N1-4 (HMT) and relevant mutant strains using qRT-PCR. **Figure S4.** Transcriptional analyses of *qssR1*, *qssR2*, *qssR3*, *qssR4* and *qssR5* in wild-type N1-4 (HMT) and relevant mutant strains using qRT-PCR. **Figure S5.** Transcriptional analyses of *qssR1*, *qssR2*, *qssR3*, *qssR4* and *qssR5* in wild-type N1-4 (HMT) and relevant mutant strains using qRT-PCR. **Figure S6.** Transcriptional analyses of *qssP1*, *qssP2*, *qssP3*, *qssP4* and *qssP5* in wild-type N1-4 (HMT) and relevant mutant strains using qRT-PCR. **Figure S7.** Transcriptional analyses of *qssP1*, *qssP2*, *qssP3*, *qssP4* and *qssP5* in wild-type N1-4 (HMT) and relevant mutant strains using qRT-PCR. **Figure S8.** Transcriptional analyses of *qssP1*, *qssP2*, *qssP3*, *qssP4* and *qssP5* in wild-type N1-4 (HMT) and relevant mutant strains using qRT-PCR. **Figure S9.** Transcriptional analyses of *spo0E*-like genes in wild-type N1-4 (HMT) and relevant mutant strains using qRT-PCR.

## Data Availability

Not applicable.

## Abbreviations

ABE: Acetone–butanol–ethanol
QSSs: Quorum-sensing systems
Opp: Oligopeptide permease
TPRs: Tetratricopeptide repeats
AIP: Auto-inducing peptide
CRISPR: Clustered Regularly Interspaced Short Palindromic Repeats
TGY: Tryptone–glucose–yeast extract
qRT-PCR: Quantitative reverse transcription PCR
cPCR: Colony PCR
