# Supplementary material for: RRNPP-type quorum-sensing systems regulate solvent formation, sporulation and cell motility in Clostridium saccharoperbutylacetonicum
Source: Biotechnol Biofuels. 2020 May 8;13:84. doi: 10.1186/s13068-020-01723-x (PMC7206700; doi:10.1186/s13068-020-01723-x)
Supplement: Supplementary file 1 — Additional file 1: Table S1. Primers used in this study. Table S2. Comparative pair-alignment data of amino acid sequences of the Clostridium saccharoperbutylacetonicum RRNPP-type transcriptional regulators. Table S3. Comparative pair-alignment data of amino acid sequences of the signaling-peptide precursors of the five Clostridium saccharoperbutylacetonicum RRNPP-type quorum sensing systems. Figure S1. Schematic of two rounds of PCR to obtain the DNA fragment containing 20-nt gRNA sequence for constructing the CRISPR–Cas9 plasmid for gene deletion. Figure S2. Confirmation of gene deletion by colony PCR. Figure S3. Transcriptional analyses of qssR1, qssR2, qssR3, qssR4 and qssR5 in wild-type N1-4 (HMT) and relevant mutant strains using qRT-PCR. Figure S4. Transcriptional analyses of qssR1, qssR2, qssR3, qssR4 and qssR5 in wild-type N1-4 (HMT) and relevant mutant strains using qRT-PCR. Figure S5. Transcriptional analyses of qssR1, qssR2, qssR3, qssR4 and qssR5 in wild-type N1-4 (HMT) and relevant mutant strains using qRT-PCR. Figure S6. Transcriptional analyses of qssP1, qssP2, qssP3, qssP4 and qssP5 in wild-type N1-4 (HMT) and relevant mutant strains using qRT-PCR. Figure S7. Transcriptional analyses of qssP1, qssP2, qssP3, qssP4 and qssP5 in wild-type N1-4 (HMT) and relevant mutant strains using qRT-PCR. Figure S8. Transcriptional analyses of qssP1, qssP2, qssP3, qssP4 and qssP5 in wild-type N1-4 (HMT) and relevant mutant strains using qRT-PCR. Figure S9. Transcriptional analyses of spo0E-like genes in wild-type N1-4 (HMT) and relevant mutant strains using qRT-PCR. [file 13068_2020_1723_MOESM1_ESM.docx]

**Additional file 1**

**RRNPP-type quorum sensing systems regulate solvent formation, sporulation and cell motility in *Clostridium saccharoperbutylacetonicum***

Jun Feng^a^, Wenming Zong^a,b^, Pixiang Wang^a^, Zhong-Tian Zhang^a^, Yanyan Gu^a^, Mark Dougherty^a^, Ilya Borovok ^c,*^, Yi Wang^a,d,*^

^a^Department of Biosystems Engineering, Auburn University, Auburn, AL 36849, USA

^b^School of Engineering, Anhui Agricultural University, Hefei 230036, China

^c^Department of Molecular Microbiology and Biotechnology, Tel Aviv University, Ramat Aviv, Israel

^d^Center for Bioenergy and Bioproducts, Auburn University, Auburn, AL 36849, USA

***Corresponding author:**

Yi Wang,

Department of Biosystems Engineering,

Auburn University,

215 Tom E. Corley Building,

Auburn, AL, 36849 USA

Tel: 1-334-844-3503; Fax: 1-334-844-3530;

E-mail: [yiwang3@auburn.edu](mailto:yiwang3@auburn.edu)

Ilya Borovok,

Department of Molecular Microbiology and Biotechnology,

Tel Aviv University,

Ramat Aviv, 6997801 Tel Aviv, Israel

Tel: 972-52-8665-901; Fax: 972-3-640-9407;

E-mail: ilyabo@tauex.tau.ac.il

**Methods**

**Plasmid construction**

The names and sequences of all the primers used in this study are listed in Table S1. In this study, the RRNPP-type regulator genes *Cspa_c00280*, *Cspa_c21720*, *Cspa_c27220*, *Cspa_c29260* and *Cspa_c56960* were named as *qssR1*, *qssR2*, *qssR3*, *qssR4* and *qssR5*, respectively (Fig. 1); their putative cognate signaling peptide genes *Cspa_c00290*, *Cspa_c21710*, *Cspa_c27230*, *Cspa_c29250* and *Cspa_c56950* were named as *qssP1*, *qssP2*, *qssP3*, *qssP4* and *qssP5*, respectively (Fig. 1); the five putative RRNPP-type systems were named as QSS1 to QSS5.

The Phanta Max Super-Fidelity DNA Polymerase (Vazyme Biotech Co., Ltd., Nanjing, China) was used for the PCR to amplify DNA fragments for cloning purposes. For gene deletion, the plasmid pYW34, which contains a lactose-inducible promoter driving the expression of the *Streptococcus pyogenes* *cas9* gene along with a chimeric gRNA sequence, was used as the mother vector ^1, 2^. The synthetic J23119 promoter and the gRNA containing the 20-nt guide sequence were amplified by two rounds of PCR with primers N-20nt/YW1342 and YW1339/YW1342 as described previously (as a naming rule of this study, N represents the corresponding gene name: *qssR1*, *qssR2*, *qssR3*, *qssR4* and *qssR5*) (Fig. S1) ^2^. The obtained fragments were then inserted into pYW34 (digested with *Btg*ZI and *Not*I) through Gibson Assembly. The two 500-bp homologous arms were ligated into the above obtained plasmids (digested with *Not*I), generating the plasmid pYW34-*N* for the deletion of gene N.

To construct pYW19d-*qssR4*, the J23119 promoter and the gRNA with 20-nt guide sequence were amplified by two rounds of PCR with primers *qssR4*-20nt/YW1342 and YW1339/YW1342. The obtained fragment was then inserted into pYW19d-*BseR*I plasmid (digested with *Bse*RI and *Not*I) through Gibson Assembly ^3^.

To construct the plasmid for genetic complementation, the promoter of the *cat1* gene (CTK_C06520) from *C. tyrobutyricum* ATCC 25755 and the open reading frames of *qssR1*, *qssR2*, *qssR3*, *qssR4* or *qssR5* were inserted through Gibson Assembly into pMTL82151 (digested with *Eco*RI and *Kpn*I), respectively, generating pMTL-*qssR1*, pMTL-*qssR2*, pMTL-*qssR3*, pMTL-*qssR4*, and pMTL-*qssR5*.

**Plasmid transformation and mutant identification**

*C. saccharoperbutylacetonicum* competent cells were prepared following the procedure as described by Herman et al. with modifications ^4^. Briefly, when the optical density (OD_600_) of the cell culture reached ~0.8, cells were harvested by centrifugation at 4,200 g, 22 °C for 10 min. The supernatant was discarded (operated in the anaerobic chamber) and cell pellets were then resuspended in the same volume of SMP buffer (270 mM sucrose, 1 mM MgCl_2_ and 7 mM sodium phosphate). The resuspension was centrifuged again under the same conditions as described above. The supernatant was discarded, and the cell pellets were resuspended by 1/20 volume of SMP buffer. After that, the plasmid (~1.0 μg) was mixed with 400 μL of competent cells and the mixture was transferred into a 2 mm electroporation cuvette followed by incubation in ice for 30 min. Electroporation was then conducted using a Gene Pulser Xcell electroporation system (Bio-Rad Laboratories, Hercules, CA) with following parameters: voltage of 1,000 V, capacitance of 25 μF and resistance of 300 Ω. Subsequently, the culture was transferred into 2 mL pre-warmed TGY medium and incubated at 35 °C for 2-3 h. After that, the culture was spun down and spread onto TGYC plates (TGY agar plate containing 30 μg/mL clarithromycin) or TGYT plates (TGY agar plate containing 15 μg/mL thiamphenicol).

The positive gene deletion mutant was screened as previously described ^2^. Briefly, the transformant harboring the CRISPR-Cas9 plasmid for gene deletion was incubated in TGYC liquid medium at 35 °C for about 12 h. The cell culture was then spread onto TGYLC plates (TGYC supplemented with 40 mM lactose). When colonies were observed, colony PCR (cPCR) was then performed with primer pairs of N-U/N-D (N represents the gene names: *qssR1*, *qssR2*, *qssR3*, *qssR4* and *qssR5*), to verify the gene deletion mutation (Table S1). The verified mutant was then subcultured in TGY liquid medium for 3-5 generations to cure the plasmid ^2^. The finally obtained plasmid-free mutant was designated as *C. saccharoperbutylacetonicum* ∆R1, ∆R2, ∆R3, and ∆R5 (the deletion of *qssR4* was unsuccessful despite numerous attempts).

**Quantitative reverse transcription PCR (qRT-PCR)**

For the qRT-PCR analysis of genes associated with cell motility (*fliA* and *flgC*), the cell culture was grown in TGY medium for 12 h and then cells were harvested for the analysis. For the qRT-PCR analysis of *qssR1*, *qssR2*, *qssR3*, *qssR4*, *qssR5*, *qssP1*, *qssP2*, *qssP3*, *qssP4*, *qssP5*, *sol* operon (*bld* as the first gene of the *sol* operon was analyzed) and genes associated with sporulation including *spo0A* (*Cspa_c27540*) and *spo0E*-like *genes* (*Cspa_c00300*, *Cspa_c21700* and *Cspa_c56940*), the cell culture was grown in TGY medium for 12 h and then grown in P2 medium (with 80 g/L glucose, 2 g/L yeast extract, and 6 g/L tryptone) or PG medium for 24 h before cells were harvested for the analysis ^5^. The seven *spo0E*-like genes *Cspa_c00300*, *Cspa_c00310*, *Cspa_c21700*, *Cspa_c29630*, *Cspa_c41910*, *Cspa_c56930*, and *Cspa_c56940* were renamed as *spo0E1-spo0E7* in this work.

The cells were collected by centrifugation at 12,000 rpm and 4 °C for 1 min. RNA was extracted using the RNA Isolater Total RNA Extraction Reagent (Vazyme Biotech Co., Ltd., Nanjing, China) following the manufacturer’s instruction. Then, cDNA was synthesized using the HiScript II 1st Strand cDNA Synthesis Kit (Vazyme Biothech Co., Ltd, Nanjing, China). The qRT-PCR analysis was performed in technical triplicate for each cDNA sample using the Power SYBR^®^ Green PCR Master Mix (Thermofisher, Waltham, MA). The gene transcription levels were normalized against the expression of 16S rRNA gene (as the control). The results were standardized as fold changes compared to the control parental strain.

**Table S1** Primers used in this study.

| **Primers** | **Sequence (5' to 3')** |
| --- | --- |
| YW1339 | AAAGTTAAAAGAAGAAAATAGAAATTTGACAGCTAGCTCAGTCCTAGGTATAATGCTAGC |
| YW1342 | GGATCCACTAGTAACCATCACACTGGCGGCCGCTAATTATTAGTCATATCACAAAGAAT |
| *qssR1*-UF | TGATATGACTAATAATTAGCGGCCGCATCTGATTGTATTTGAATAAATTCG |
| *qssR1*-UR | CTAGCTGTTATACGATTCATTATCAAACATTGTTATTTCC |
| *qssR1*-DF | ATGTTTGATAATGAATCGTATAACAGCTAGTATTATGTTACA |
| *qssR1*-DR | ACTAGTAACCATCACACTGTGTTCTACAATAGATATTTGCTCTG |
| *qssR1*-20nt | GCTCAGTCCTAGGTATAATGCTAGC*ATTAGCAGGAAGATATGCCA*GTTTTAGAGCTAGAAATAGCAAG |
| *qssR1*-U | ACTTGATTATTATGGTAGCTTGTGA |
| *qssR1*-D | GGACTTTTCCCATATTTTATCATTT |
| *qssR2*-UF | TGATATGACTAATAATTAGCGGCCGCTTAGTTTTTCATCATCAGTCACTCT |
| *qssR2*-UR | ATGTTTGATAATGAACATTATATTTTCTAATATTATGCTA |
| *qssR2*-DF | TTAGAAAATATAATGTTCATTATCAAACATTGTTATAGCC |
| *qssR2*-DR | ACTAGTAACCATCACACTGATCATCTACTGCTTCAACATATTCA |
| *qssR2*-20nt | GCTCAGTCCTAGGTATAATGCTAGC*GGAAAGATTCCTGTAAGTCA*GTTTTAGAGCTAGAAATAGCAAG |
| *qssR2*-U | GCAATATTACTAATGCTATTGTTTC |
| *qssR2*-D | TACTAACAAGCCCCTCAAATAAATC |
| *qssR3*-UF | TGATATGACTAATAATTAGCGGCCGCTATTTCTAATGTTTTTAGGATGGAG |
| *qssR3*-UR | TTAAAAATTCTTTTCTTGGTTGTCAAACATTTTATTTTCTC |
| *qssR3*-DF | ATGTTTGACAACCAAGAAAAGAATTTTTAATGTTTTTTATCG |
| *qssR3*-DR | ACTAGTAACCATCACACTGTTGAAAATAAATAAAAATCAAGTGG |
| *qssR3*-20nt | GCTCAGTCCTAGGTATAATGCTAGC*GGCCAAGAAATACCTTTTGC*GTTTTAGAGCTAGAAATAGCAAG |
| *qssR3*-U | GTATAAAAGCTTTGAGGGGGATAAT |
| *qssR3*-D | TTATTATTTTTTGCTCGGATTACCT |
| *qssR4*-UF | TGATATGACTAATAATTAGCGGCCGCAAGGTTTACCATATGCGTTGTAGTT |
| *qssR4*-UR | ATGAAATTCTTAACTAACTATATTTCATAATAATTTAGATT |
| *qssR4*-DF | TTATGAAATATAGTTAGTTAAGAATTTCATTTTAACCACC |
| *qssR4*-DR | ACTAGTAACCATCACACTGCGCAAACTCTATTGTTTTCCATTAT |
| *qssR4*-20nt | GCTCAGTCCTAGGTATAATGCTAGC*CCTCATAGCAGTTCGCTTTA*GTTTTAGAGCTAGAAATAGCAAG |
| *qssR4*-U | AAAAATGTTTTGCAATATCTAAATC |
| *qssR4*-D | TTACTGCTGAAACTAAAGAAGCATC |
| *qssR5*-UF | TGATATGACTAATAATTAGCGGCCGCATCTGATTGTATTTGAATAAATTCG |
| *qssR5*-UR | CTAGCTGTTATACGATTCATTATCAAACATTGTTATTTCC |
| *qssR5*-DF | ATGTTTGATAATGAATCGTATAACAGCTAGTATTATGTTACA |
| *qssR5*-DR | ACTAGTAACCATCACACTGTGTTCTACAATAGATATTTGCTCTG |
| *qssR5*-20nt | GCTCAGTCCTAGGTATAATGCTAGC*TCATCTATCATTAATTCCCC*GTTTTAGAGCTAGAAATAGCAAG |
| *qssR5*-U | ACTTGATTATTATGGTAGCTTGTGA |
| *qssR5*-D | GGACTTTTCCCATATTTTATCATTT |
| *qssR1*-F | ATATAATTTATGAAAGGGTGGTTTTTATGTTTGATAATGAAATCTTGACGC |
| *qssR1*-R | CGACTCTAGAGGATCCCCGGGTACCGAATTCAACATAATACTAGCTGTTATACC |
| *qssR2*-F | ATATAATTTATGAAAGGGTGGTTTTTATGTTTGATAATGAAATCATGATGC |
| *qssR2*-R | CGACTCTAGAGGATCCCCGGGTACCGAATTCCTTCATTTAACATTATAGCATAA |
| *qssR3*-F | ATATAATTTATGAAAGGGTGGTTTTTATGTTTGACAACCAAGTTATGCC |
| *qssR3*-R | CGACTCTAGAGGATCCCCGGGTACCGAATTCCATAACTATCCTCCTGTAAATGT |
| *qssR4*-F | ATATAATTTATGAAAGGGTGGTTTTTATGAAATTCTTAACTAATGGTCAA |
| *qssR4*-R | CGACTCTAGAGGATCCCCGGGTACCGAATTCTCTAAATTATTATGAAATATAGT |
| *qssR5*-F | ATATAATTTATGAAAGGGTGGTTTTTATGTTTAATAATGAAATTATGAAAC |
| *qssR5*-R | CGACTCTAGAGGATCCCCGGGTACCGAATTCTTCTGCCTTTACAGTGTAACAT |
| QR1-F | CTAATGCTTAGAGCAACCCAAAC |
| QR1-R | TGCTTGCTCATAGTCTTCTATCAA |
| QP1-F | GCAACATCAATGTTATTAACAAATG |
| QP1-R | TAATGTGGATCTATTGAATTTACACT |
| QE1-F | TGAGGAAATTAAAATTGAAAAATGATG |
| QE1-R | TAAATTTTGACTTGCAATTACCACC |
| QR2-F | GCTTTATCTGCCATCAGCATTA |
| QR2-R | CAGCAGCCATGTATATCAAAGAA |
| QP2-F | GATATACATTATTTGGATCCACGCT |
| QP2-R | TTATATTGGCAGTAATGGCAACATC |
| QE2-F | TAGTTGCTCTTTGTATGCAGCAATG |
| QE2-R | GTGAGAATAATGGATGATTTAATATCATA |
| QR3-F | AGTTGAACTTGCAGAAGGTATTTG |
| QR3-R | TCCGCTTCATCGAGCATTT |
| QP3-F | ATGGTCGCATGTGTTGTGTTATTAT |
| QP3-R | GATGAAACTTTTCTCCACTGCTTGA |
| QR4-F | TCCTAGTGGAGGAATATCAGATGTA |
| QR4-R | AGATCGTTATACAACGGCTCAAA |
| QP4-F | TTAGCCCCATGGAGCACTTTC |
| QP4-R | AGGCGTATCTCTATTTTTATCTTGCT |
| QR5-F | ACATCTTCATTTACGTGTGTAAGC |
| QR5-R | ATGATGTATGCGAATTGTCTAACTG |
| QP5-F | ATGGGTCAGTTGTGTTTAAATGATAC |
| QP5-R | TAGCAGTAATAGCAACGTCAATGTTA |
| QE5-F | TAAATTTTGACTTGCAATTACCACC |
| QE5-R | CGAACACCAGTTTGTGTTTGGA |
| QSol-F | CTACTGCTTGGTCAGGAGATAAC |
| QSol-R | CTCAGGACCACCACATGAAATA |
| QSpo0A-F | GGCGTTCCAGCTCATATTAAAG |
| QSpo0A-R | AGCATCTATTTGTCCTCTACCC |
| Q16S-F | CAATGGCTGGTACAGAGAGATG |
| Q16S-R | TCTCCTACGGCTACCTTGTT |
| QFliA-F | TCCAAGGAGACCATAGAAATGTAG |
| QFliA-R | GCAATGATAGACGAACTGAGAAAG |
| QFlgC-F | CCACCATCATATCTGCCATTTC |
| QFlgC-R | GCGGAAAGATTGAGAATGGATAC |

*The sequences underlined in italic are the guide sequences.

**Table S2** Comparative pair-alignment data of amino acid sequences of the *Clostridium saccharoperbutylacetonicum* RRNPP-type transcriptional regulators. ^1^

|  | QssR1 | QssR2 | QssR3 | QssR4 | QssR5 |
| --- | --- | --- | --- | --- | --- |
| QssR1 |  | 68.8% | 43.4% | 20.0% | 60.2% |
| QssR2 |  |  | 43.4% | 19.4% | 60.1% |
| QssR3 |  |  |  | 19.3% | 46.7% |
| QssR4 |  |  |  |  | 19.5% |

^1^ Only percentages of identical amino acids are shown. The results were obtained using the online ClustalW server (https://npsa-prabi.ibcp.fr/cgi-bin/npsa_automat.pl?page=/NPSA/npsa_clustalw.html).

**Table S3** Comparative pair-alignment data of amino acid sequences of the signaling-peptide precursors of the five *Clostridium saccharoperbutylacetonicum* RRNPP-type quorum sensing systems.^1^

|  | QssP1 | QssP2 | QssP3 | QssP4 | QssP5 |
| --- | --- | --- | --- | --- | --- |
| QssP1 |  | 67.9% | 38.9% | 20.0% | 69.8% |
| QssP2 |  |  | 43.6% | 25.9% | 79.3% |
| QssP3 |  |  |  | 35.6% | 43.6% |
| QssP4 |  |  |  |  | 24.1% |

^6^ Only percentages of identical amino acids are shown. The results were obtained using the online ClustalW server (https://npsa-prabi.ibcp.fr/cgi-bin/npsa_automat.pl?page=/NPSA/npsa_clustalw.html).


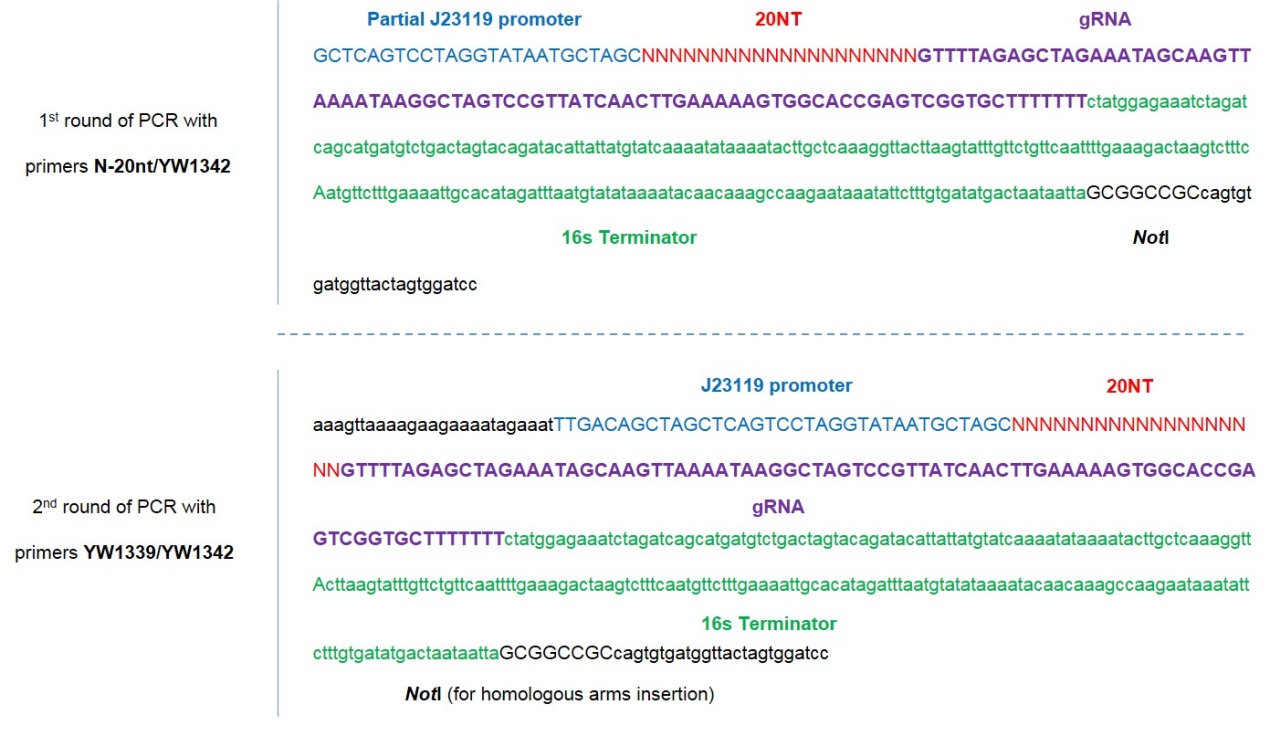


**Figure S1. Schematic of two rounds of PCR to obtain the DNA fragment containing 20-nt gRNA sequence for constructing the CRISPR-Cas9 plasmid for gene deletion**. The two rounds of PCR were used to amplify the DNA fragment to incorporate the J23119 promoter ^2^ and the 20-nt sequence. The obtained DNA fragment was then assembled into pYW34 at the *Btg*ZI and *Not*I sites.


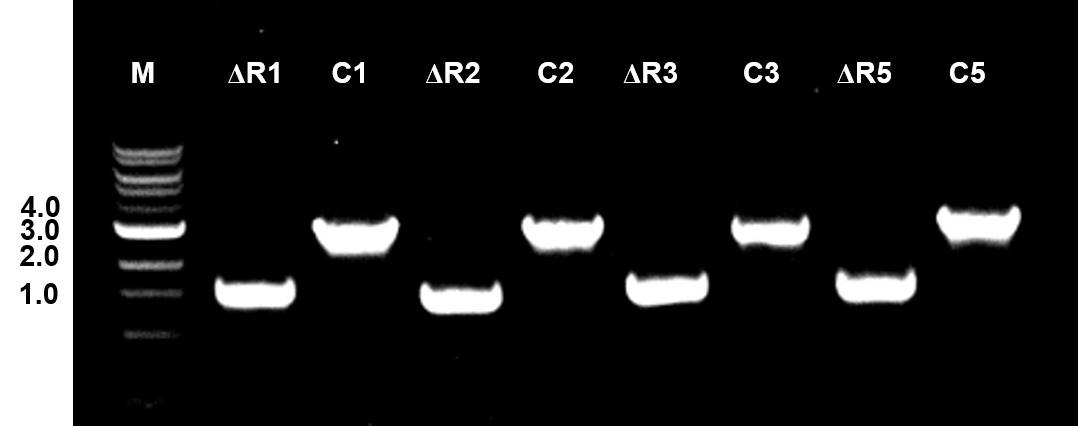


**Figure S2. Confirmation of gene deletion by colony PCR.** Lane M: The NEB 1-kb DNA ladder, with numbers on the left representing the band length in kb; Lane ΔR1: the PCR result (1,519 bp) confirming the deletion of R1 with primers *qssR1*-U/*qssR*-D. Lane C1: the PCR result (2,711 bp) from the control N1-4 (HMT) strain with primers *qssR1*-U/*qssR1*-D (2,711 bp). Lane ΔR2: the PCR result (1,394 bp) confirming the deletion of R2 with primers *qssR2*-U/*qssR2*-D. Lane C2: the PCR result (2,695 bp) from the control N1-4 (HMT) strain with primers *qssR2*-U/*qssR2*-D. Lane ΔR3: the PCR result (1,517 bp) confirming the deletion of R3 with primers *qssR3*-U/*qssR3*-D. Lane C3: the PCR result (2,774 bp) from the control N1-4 (HMT) strain with primers *qssR3*-U/*qssR3*-D. Lane ΔR5: the PCR result (1,455 bp) confirming the deletion of R5 with primers *qssR5*-U/*qssR5*-D. Lane C5: the PCR result (2,631 bp) from the control N1-4 (HMT) strain with primers *qssR5*-U/*qssR5*-D.


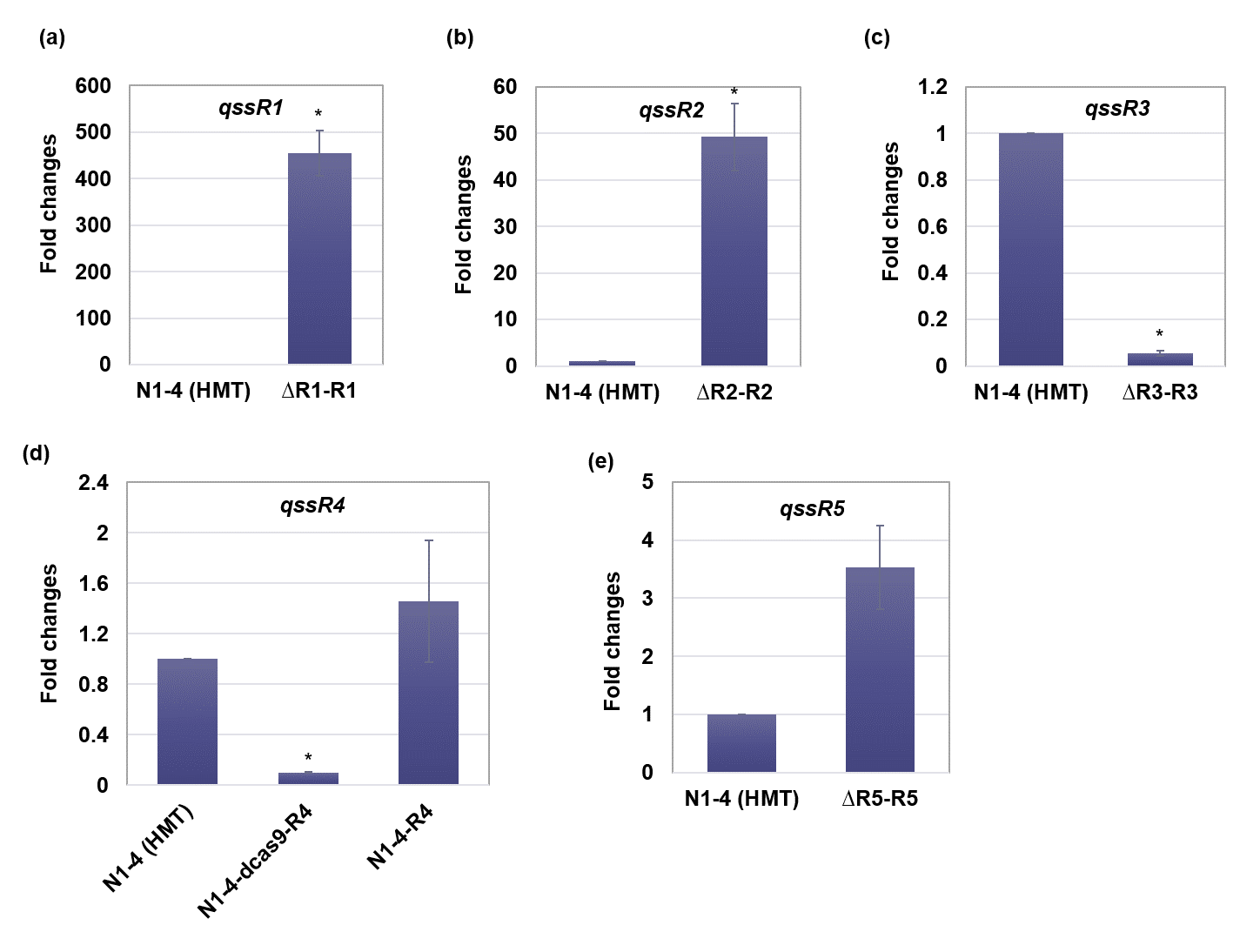


**Figure S3 Transcriptional analyses of *qssR1*, *qssR2*, *qssR3*, *qssR4* and *qssR5* in wild-type N1-4 (HMT) and relevant mutant strains using qRT-PCR**. The bacterial RNA was extracted from the cell culture after 24 h cultivation in P2 medium (see Materials and Methods). The reported value is mean ± SD. The asterisk indicates that the corresponding gene expression level in that particular strain was significantly different from the wild-type strain (P < 0.05).


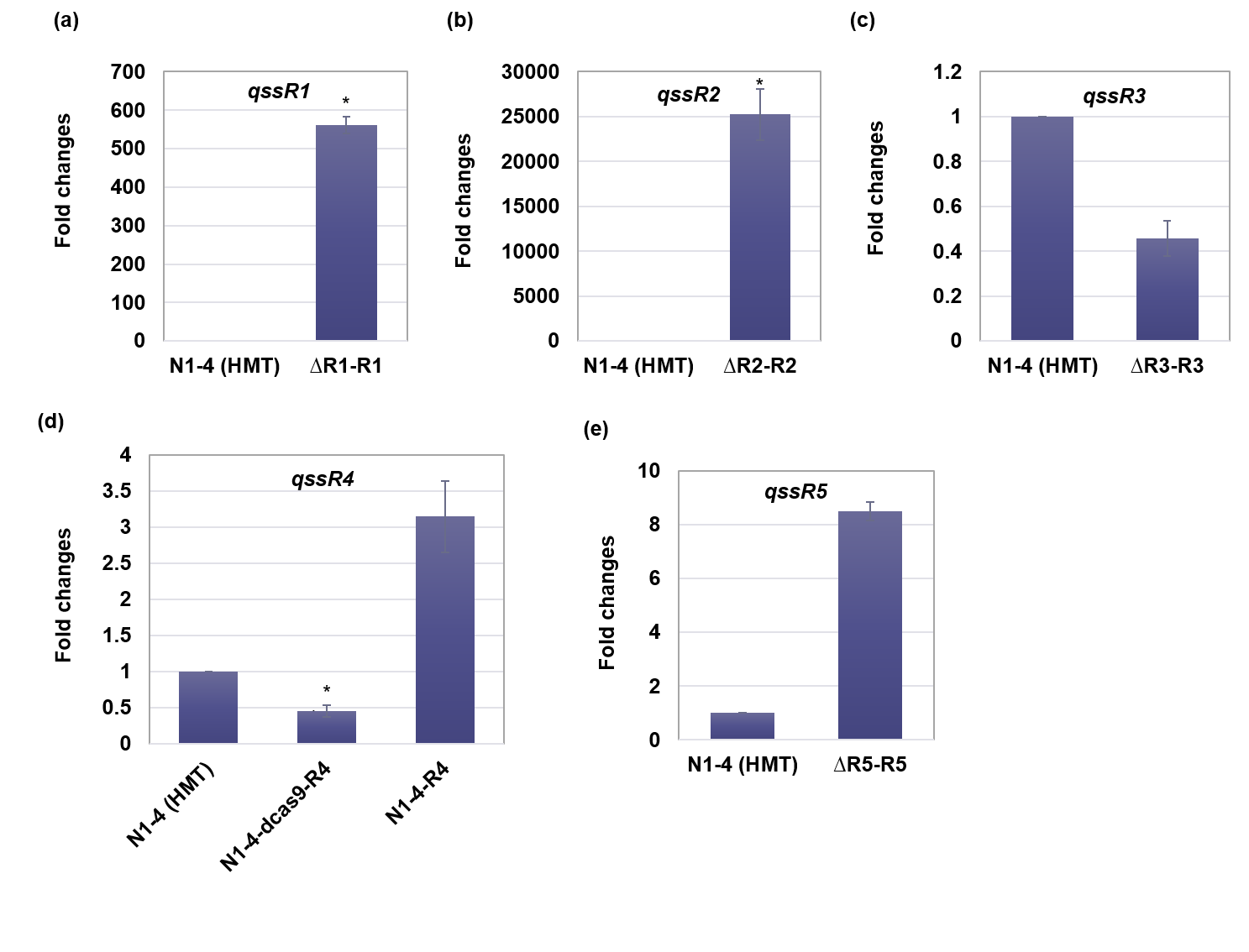


**Figure S4 Transcriptional analyses of *qssR1*, *qssR2*, *qssR3*, *qssR4* and *qssR5* in wild-type N1-4 (HMT) and relevant mutant strains using qRT-PCR**. The bacterial RNA was extracted from the cell culture after 24 h cultivation in TGY medium (see Materials and Methods). The reported value is mean ± SD. The asterisk indicates that the corresponding gene expression level in that particular strain was significantly different from the wild-type strain (P < 0.05).


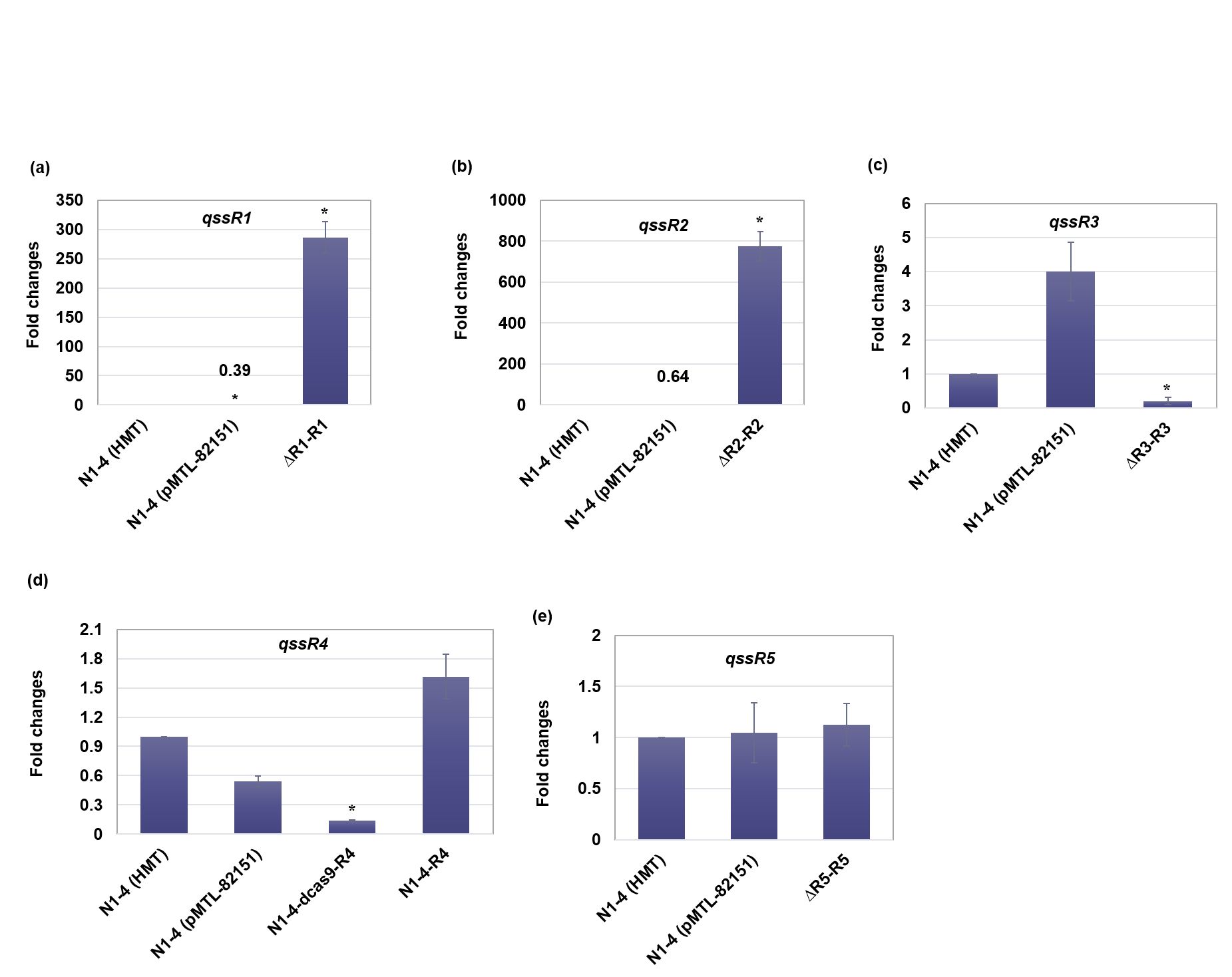


**Figure S5 Transcriptional analyses of *qssR1*, *qssR2*, *qssR3*, *qssR4* and *qssR5* in wild-type N1-4 (HMT) and relevant mutant strains using qRT-PCR**. The bacterial RNA was extracted from the cell culture after 24 h cultivation in PG medium (see Materials and Methods). The reported value is mean ± SD. The asterisk indicates that the corresponding gene expression level in that particular strain was significantly different from the wild-type strain (P < 0.05).


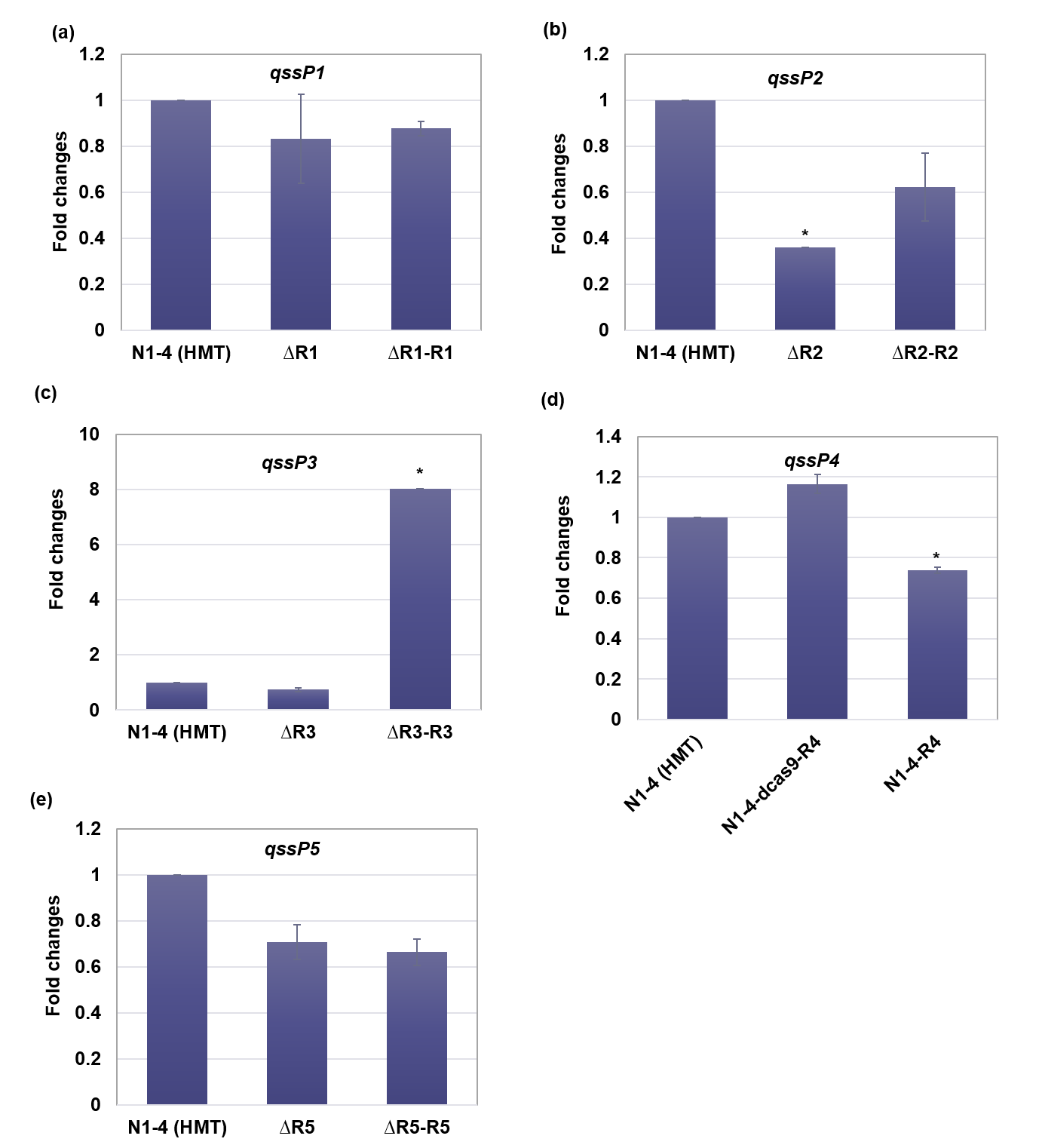


**Figure S6 Transcriptional analyses of *qssP1*, *qssP2*, *qssP3*, *qssP4* and *qssP5* in wild-type N1-4 (HMT) and relevant mutant strains using qRT-PCR**. The bacterial RNA was extracted from the cell culture after 12 h cultivation in TGY medium (see Materials and Methods). The reported value is mean ± SD. The asterisk indicates that the corresponding gene expression level in that particular strain was significantly different from the wild-type strain (P < 0.05).


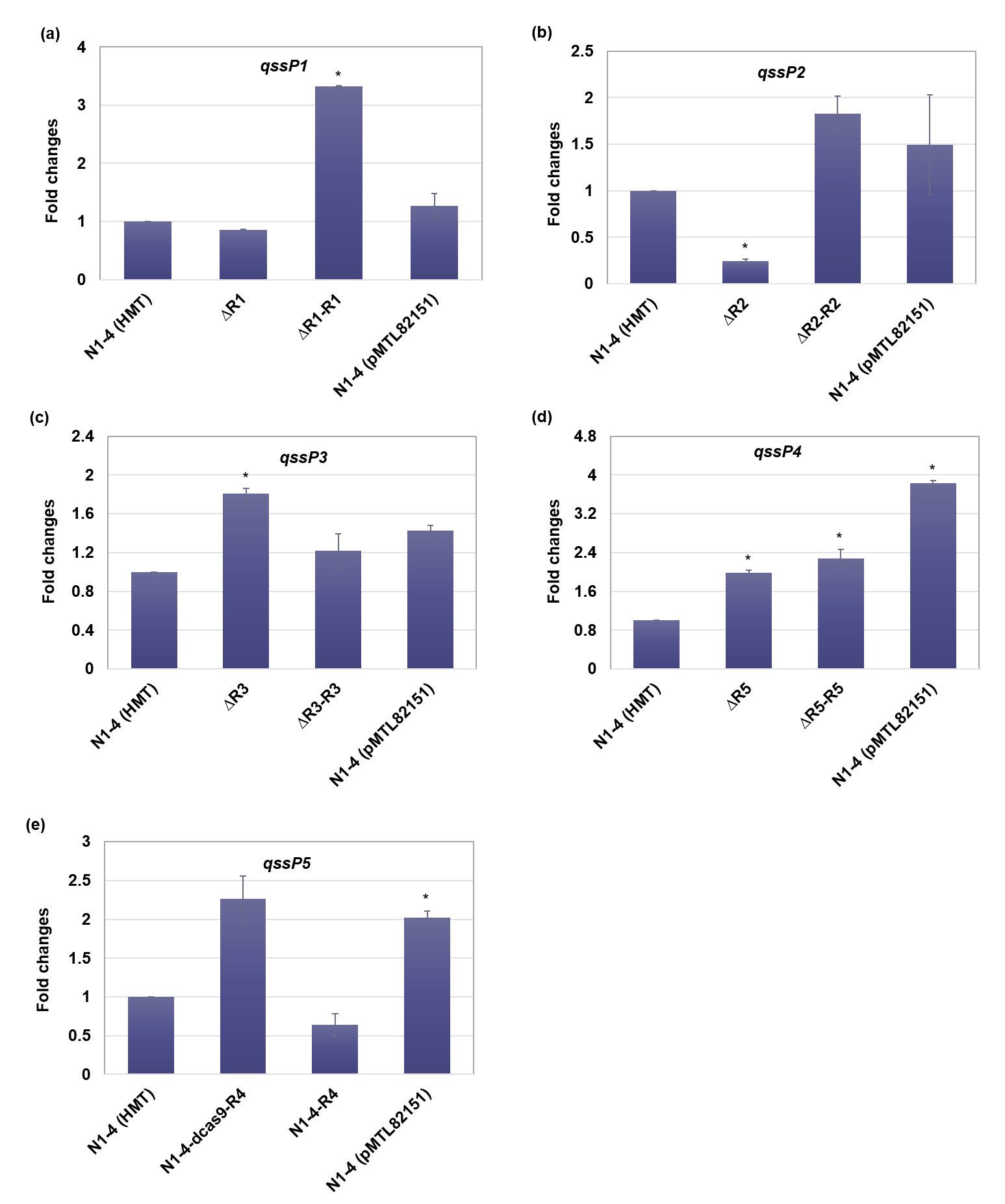


**Figure S7 Transcriptional analyses of *qssP1*, *qssP2*, *qssP3*, *qssP4* and *qssP5* in wild-type N1-4 (HMT) and relevant mutant strains using qRT-PCR**. The bacterial RNA was extracted from the cell culture after 24 h cultivation in PG medium (see Materials and Methods). The reported value is mean ± SD. The asterisk indicates that the corresponding gene expression level in that particular strain was significantly different from the wild-type strain (P < 0.05).


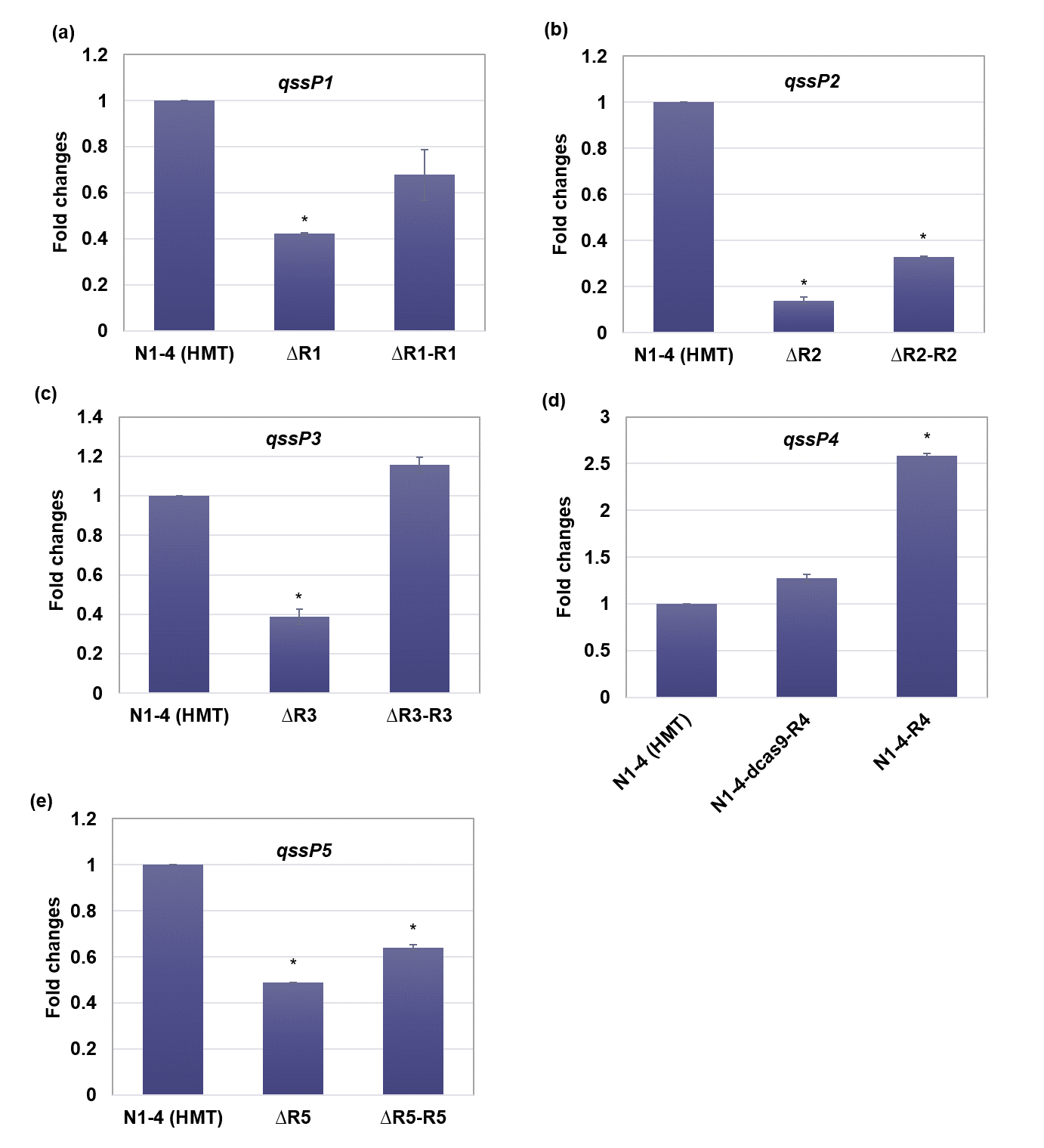


**Figure S8 Transcriptional analyses of *qssP1*, *qssP2*, *qssP3*, *qssP4* and *qssP5* in wild-type N1-4 (HMT) and relevant mutant strains using qRT-PCR**. The bacterial RNA was extracted from the cell culture after 24 h cultivation in P2 medium (see Materials and Methods). The reported value is mean ± SD. The asterisk indicates that the corresponding gene expression level in that particular strain was significantly different from the wild-type strain (P < 0.05).


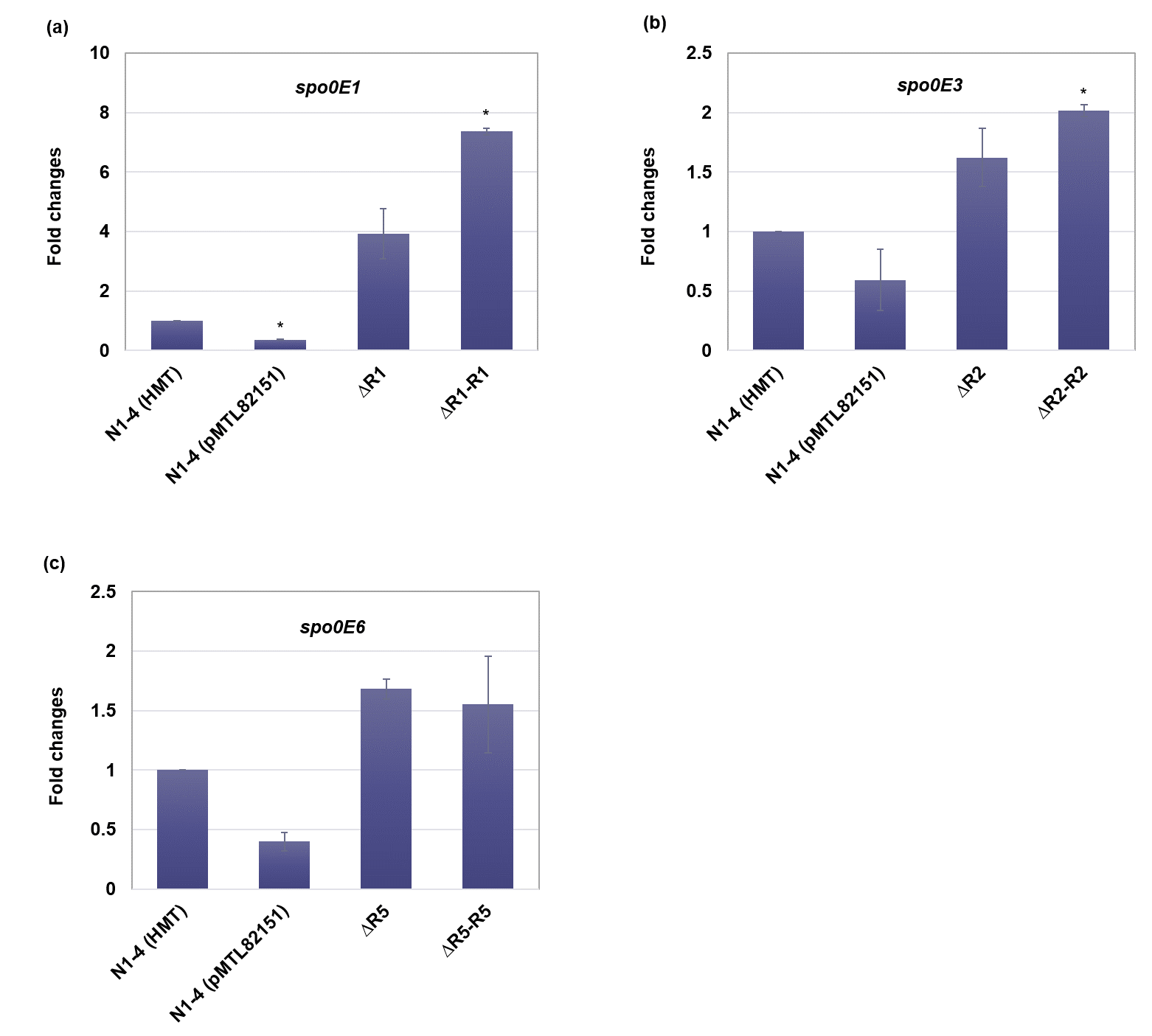


**Figure S9 Transcriptional analyses of *spo0E*-like genes in wild-type N1-4 (HMT) and relevant mutant strains using qRT-PCR**. The bacterial RNA was extracted from the cell culture after 24 h cultivation in PG medium (see Materials and Methods). The reported value is mean ± SD. The asterisk indicates that the corresponding gene expression level in that particular strain was significantly different from the wild-type strain (P < 0.05).

**References:**

1. Wang, Y. et al. Bacterial genome editing with CRISPR-Cas9: deletion, Integration, single nucleotide modification, and desirable “clean” mutant selection in *Clostridium beijerinckii* as an example. *ACS synthetic biology* **5**, 721-732 (2016).

2. Wang, S., Dong, S., Wang, P., Tao, Y. & Wang, Y. Genome Editing in Clostridium saccharoperbutylacetonicum N1-4 with the CRISPR-Cas9 System. *Applied and Environmental Microbiology* **83**, e00233-00217 (2017).

3. Wang, Y. et al. Gene transcription repression in *Clostridium beijerinckii* using CRISPR-dCas9. *Biotechnology and bioengineering* **113**, 2739-2743 (2016).

4. Herman, N.A. et al. Development of a High-Efficiency Transformation Method and Implementation of Rational Metabolic Engineering for the Industrial Butanol Hyperproducer *Clostridium saccharoperbutylacetonicum* Strain N1-4. *Applied and environmental microbiology* **83**, e02942-02916 (2017).

5. Wang, S., Dong, S. & Wang, Y. Enhancement of solvent production by overexpressing key genes of the acetone-butanol-ethanol fermentation pathway in Clostridium saccharoperbutylacetonicum N1-4. *Bioresource technology* **245**, 426 (2017).

6. Thompson JD, Higgins DG. Gibson TJ. CLUSTAL W: improving the sensitivity of progressive multiple sequence alignment through sequence weighting, position-specific gap penalties and weight matrix choice. 1994;22:4673-4680.
